# Pharmacological manipulation of Sema4D by salvianolic acid A mitigates diabetic retinopathy via inhibiting PlexinB1/RhoA/ROCK/pMLC2 signaling cascade involved in endothelial dysfunction

**DOI:** 10.1186/s13020-026-01468-z

**Published:** 2026-07-20

**Authors:** Weiwei Zheng, Ling Ning, Peiliang Shen, Jing Ma, Chang Yu, Ruiqin Jia, Liwenyu Chen, Wei Zou, Yuhua Xu, Yanhong Pan, Zhonghong Wei, Qiuhong Shen, Chongjin Zhong, Aiyun Wang, Wenxing Chen, Juan Chen, Suyun Yu, Jia Li, Yin Lu, Yang Zhao

**Affiliations:** 1 Jiangsu Key Laboratory for Pharmacology and Safety Research of Chinese Materia Medica, Nanjing University of Chinese Medicine, 138 Xianlin Avenue, Nanjing, 210023 China; 2 Department of Biochemistry and Molecular Biology, School of Medicine, Nanjing University of Chinese Medicine, Nanjing, 210023 China; 3 School of Pharmacy, Henan University, Kaifeng, 450046 China; 4 Jiangsu Health Vocational College, Nanjing, 211800 China; 5 The Affiliated Huaian No.1 People’s Hospital of Nanjing Medical University, Huaian, 223300 China; 6 Cutin Medical School, Curtin Medical Research Institute, Curtin University, Curtin MRI, Building 305, Bentley, WA U19887 Australia; 7 School of Diagnostic and Therapeutic Sciences, Curtin University, Bentley, WA U19887 Australia; 8 Perron Institute for Neurological and Translational Research, Nedlands, WA6009 Australia

**Keywords:** Salvianolic acid A, Diabetic retinopathy, Sema4D, Astrocytes, Endothelial junctions, Liposomes

## Abstract

**Background:**

Diabetic retinopathy (DR) is a leading cause of blindness. While anti-vascular endothelial growth factor (VEGF) therapy is effective, its utility is limited by variable patient response and the need for frequent injections. Therefore, identifying new therapeutic targets for DR is imperative. Emerging evidence indicates that astrocytes contribute to endothelial dysfunction in DR, suggesting that targeting astrocyte-endothelial cell crosstalk represents a promising therapeutic strategy.

**Purpose:**

To evaluate the therapeutic potential of Salvianolic acid A (Sal A) for DR, elucidate the molecular mechanisms by which it modulates astrocyte-endothelial cell interactions, and develop a liposome-based nanodelivery system to enhance its efficacy.

**Study Design:**

The protective effects and mechanisms of Sal A were systematically investigated using a streptozotocin (STZ)-induced diabetic mouse model, complemented by a suite of in vitro and molecular approaches including co-culture models, transcriptomic analysis, and target validation assays.

**Methods:**

Retinal vascular structure and barrier function were assessed in vivo via immunofluorescence staining and Evans Blue leakage assays. Endothelial cell behaviors were examined in vitro using wound healing, Transwell migration, tube formation, and spheroid sprouting assays. Transcriptomic profiling was performed by RNA sequencing (RNA-seq). The direct target of Sal A was identified and validated using MS-based drug-affinity responsive target stability (DARTS) screening, cellular thermal shift assay (CETSA), and microscale thermophoresis (MST). Expression of key signaling molecules was measured by western blotting, enzyme-linked immunosorbent assay (ELISA), and quantitative real-time PCR (qRT-PCR). Liposome@Sal A was prepared and characterized for its physicochemical properties (dynamic light scattering, transmission electron microscopy), stability, and therapeutic efficacy in vitro and in vivo.

**Results:**

Sal A treatment ameliorated retinal vascular abnormalities in diabetic mice, evidenced by increased VE-cadherin and NG2 expression, decreased α-smooth muscle actin (α-SMA) expression, and reduced acellular capillary formation, collectively restoring vascular integrity. Mechanistically, astrocyte-derived soluble Semaphorin 4D (sSema4D) promoted endothelial hyperactivation via the PlexinB1/RhoA/ROCK/pMLC2 signaling cascade. Sal A directly bound to the Arg92 residue of membrane-bound Sema4D on astrocytes, significantly inhibiting sSema4D shedding and its subsequent deleterious effects on endothelial cells. Furthermore, Liposome@Sal A enhanced retinal drug delivery and demonstrated superior therapeutic efficacy compared to free Sal A in diabetic mice.

**Conclusion:**

Sal A preserves retinal vascular structure and function in DR by binding to astrocytic Sema4D at Arg92, thereby inhibiting sSema4D shedding and downstream PlexinB1/RhoA/ROCK/pMLC2 signaling, which modulates astrocyte-endothelial cell crosstalk. Liposomal encapsulation significantly potentiates the therapeutic efficacy of Sal A, positioning it as a promising drug candidate for DR treatment.

**Supplementary Information:**

The online version contains supplementary material available at 10.1186/s13020-026-01468-z.

## Background

Diabetic retinopathy (DR) is one of the most frequent microvascular complication of diabetes and serves as the leading cause of blindness in the working-age population. It has been reported that approximately 103.12 million adults were diagnosed with DR worldwide in 2020, and about 160.5 million people are expected to have DR in 2045 globally with 44.82 million people undergoing vision issues [1, 2]. This rapid rise may be owing to the increasing prevalence of diabetes, aging of the population and elevation of life expectancy of those with diabetics. The majority of patients are diagnosed with DR between 10 to 15 years after the onset of their diabetes [3]. DR therefore poses significant health and socioeconomic challenges all over the world.

It has been increasingly recognized that the pathology of DR is characterized by dysfunction of endothelial cells (ECs)-lined blood vessels, which demonstrates early loss of pericytes, vascular leakage, development of acellular capillaries, and late-stage retinal ischemia, eventually leading to excessive neovascularization in retinas [4, 5]. Owing to the crucial involvement of vascular endothelial growth factor A (VEGF-A) in yielding dysfunction of ECs, intravitreal injection of its neutralizing antibody has been commonly utilized to fight against the development of diabetic macular edema (DME) and proliferative DR (PDR) in clinical settings [6–8]. Nonetheless, inhibition of VEGF-A fails to completely impede the initiation and progression of DR. A large number of patients with DR still experience deteriorations and ultimately develop blindness following the conventional clinical therapies. As such, in-depth investigating the pathogenesis of DR, identifying novel targets and seeking effective strategies for limiting the development of DR treatment have been of great clinical and social significance for alleviating the medical burden of DR patients.

Indeed, it has been widely held that numerous glial cells are presented in the retina, predominantly including microglia cells, astrocytes and Müller cells [9]. These cells have been validated to play indispensable roles in maintaining the retinal structure and function. Among them, the interactions between astrocytes and ECs have been obtaining growing attention [10–12]. The distribution of retinal astrocytes is tightly correlated to the presence and organization of retinal blood vessels, resulting in vascularized areas of the retina being abundant in astrocytes, whereas avascular regions including fovea are devoid of astrocytes [13]. Under the circumstance of injury or disease, astrocytes exert striking effects on boosting the expression of numerous genes associated with cytokines, chemokines, and components of the complement pathway. This response is deemed to provoke impaired integrity of the blood-retinal barrier (BRB) and further render retinal degeneration [14]. In this perspective, targeting the interactions between astrocytes and ECs may provide a promising therapeutic approach for hampering the progression of DR.

Notably, Medicinal plants have been widely used as an important source for preventing and managing many types of diseases in developing countries for several centuries. Plants possess the ability to synthesize a broad variety of chemical compounds, which provide a great opportunity to discover effective agents to treat diabetic retinopathy. *Salvia miltiorrhiza* Bunge (also known as Danshen) is a well-known medicinal herb that displays a wide spectrum of cardiovascular and cerebrovascular protective activities, such as powerful vascular protection, antioxidant, and neuroprotective functions [15]. Intriguingly, recent studies have pinpointed that these pharmacological properties are capable of effectively ameliorating the endothelial dysfunction correlated to the progression of DR [16]. Salvianolic acid A (Sal A), a predominant water-soluble and biologically active ingredient extracted from Danshen, has been documented to diminish oxidative injury and protect against compromised vascular responsiveness in the streptozotocin (STZ)-triggered diabetic rats. More specifically, Sal A contributes to a significant reduction in light of the maximum contraction (E(max)) to noradrenaline in the diabetic aortas [17]. However, the precise role of Sal A in influencing the development of DR is still obscure.

In the present study, a STZ-induced diabetic mouse model is employed to determine the impacts of Sal A on the development of DR. It has been demonstrated that Sal A exerts imperative effects on fighting against the progression of DR. Mechanistic investigations uncovers that Sal A is able to directly bind to the Arg92 residue of semaphorin 4D (Sema4D) on the membranes of astrocytes, resulting in prominent decrease in the shedding of soluble Sema4D (sSema4D). More significantly, Sal A reverses the disrupted function of ECs induced by astrocytes through inhibiting the activation of sSema4D/PlexinB1/RhoA/ROCK/pMLC2 signaling cascade. Moreover, liposome-encapsulated Sal A nanoparticles (liposome@Sal A NPs) are constructed to enhance the stability of Sal A. It is shown that liposome@Sal A NPs show superior therapeutic efficacy on DR compared with Sal A alone. Taken together, Sal A emerges as a promising agent for improving vascular structure and function, and it can be further optimized to be a potent drug candidate for the treatment of DR.

## Materials and methods

### Reagents

Sal A (PubChem CID: 5,281,793, catalog number: B20260) was purchased from Yuanye Bio-Technology Co., Ltd. (Shanghai, China). The primary antibodies used in this study are listed as follows: VE-Cadherin (2500S; Cell Signaling Technology, Beverly, USA), ZO-1 (13663S; Cell Signaling Technology, Beverly, USA), GFAP (sc-33673; Santa Cruz Biotechnology, CA, USA), Sema4D (53108S; Cell Signaling Technology, Beverly, USA), CD31 (557,355; BD Biosciences, NJ, USA), Isolectin B4 (ZH0604; Vector laboratories, CA, USA), TRITC Phalloidin (40734ES75; YEASEN, Shanghai, China), NG2 (Ab259324; Abcam, Cambridge, UK), α-SMA (19,245; Cell Signaling Technology, Beverly, USA), pMLC2 (3674S; Cell Signaling Technology, Beverly, USA), Claudin-5 (49564S; Cell Signaling Technology, Beverly, USA), PlexinB1 (sc-28372; Santa Cruz Biotechnology, CA, USA), RhoA (sc-418; Santa Cruz Biotechnology, CA, USA), ROCK2 (sc-398519; Santa Cruz Biotechnology, CA, USA), GAPDH (GB15004; Servicebio, Wuhan, China), Streptavidin Alexa Fluor™ 488 conjugate (S11223; Invitrogen, CA, USA), Goat anti-Rabbit IgG (H + L) Cross-Adsorbed Secondary Antibody, Alexa Fluor™ 594 (A11012; Invitrogen, CA, USA), Goat Anti-Rabbit IgG (H + L) HRP (AA86182; Bioworld, Nanjing, China), Goat Anti-Mouse IgG(H + L) HRP (BS12478; Bioworld, Nanjing, China), FITC-dextran (HY-128868A; MCE, NJ, USA), Narciclasine (HY-16563, MCE, NJ, USA), Fasudil (HY-1077, MCE, NJ, USA).

### Cell lines

The SVG p12 fetal glial cells were obtained from QINQI Biotechnology Development Co., Ltd. (Shanghai, China) and were cultured at 37℃ with 5% CO_2_ in Dulbecco's Modified Eagle's Medium (DMEM) supplemented with 10% fetal bovine serum (FBS) (Cellmax, Lanzhou, China) and 1% penicillin–streptomycin solution (Corning, NY, USA). Human cortical microvascular endothelial cells/D3 (hCMEC/D3) and 293 T cells were obtained from Procell (Wuhan, China). hCMEC/D3 and 293 T cells were maintained in DMEM supplemented with 10% FBS and 100 U/mL penicillin and streptomycin at 37 ℃ in an incubator with 5% CO_2_. Human umbilical vein ECs (HUVECs) were isolated from donated cords with approval from the Ethics Committee of Jiangsu Province Hospital on Integration of Chinese and Western Medicine (permit and approval number: 2021-LWKY-003) and were cultured in medium 199 + 20% FBS + 1% Penicillin–Streptomycin Solution + 15 µg/ml endothelial cell growth supplement (ScienCell, San Diego, USA) [18].

### Cell transfection

The siRNAs targeting PlexinB1 were generated in Corues Biotechnology (Nanjing, China). The control or PlexinB1 siRNAs were transfected into cells using the Lipofectamine 2000 Transfection Reagent (Invitrogen, CA, USA). The sequences of PlexinB1 siRNAs are showed in Table S1. The knockdown efficiency was examined by western blot analysis 24 h following cell transfection. The pCas-Puro-U6-Sema4D-KO plasmid was constructed by Corues Biotechnology (Nanjing, China). Plasmid transfection was performed using ExFect transfection reagent (Vazyme, Nanjing, China), and the knockout efficiency of Sema4D was subsequently validated by western blot analysis.

### Animal studies

All animal studies were approved by the Animal Ethics Committee of Nanjing University of Chinese Medicine (approval number: 202312A069, 202604A87). Seven-week-old male C57BL/6 J mice (license number: SCXK2022-0004) were purchased from the SLAC Laboratory Animal Co., Ltd. (Shanghai, China). The mice were starved for 4 h, after which they were intraperitoneally administered with 180 mg/kg STZ (Sigma, MO, USA) or citrate buffer (vehicle control) for 10 days consecutively [19]. The fasting blood glucose of the mice was evaluated following the last injection of STZ by a glucometer. The mice with fasting blood glucose concentrations higher than 16.7 mM were deemed to be diabetic. Subsequently, Sal A (5 mg/kg or 10 mg/kg) and Liposome@Sal A (5 mg/kg) were administered via intravenous injection once weekly for 2 weeks, whereas bevacizumab (10 mg/kg) was delivered by intravitreal injection to the diabetic mice once weekly for 2 weeks. Following completion of the treatment regimen, the retinas were harvested to assess retinopathy-associated pathological changes.

### RNA sequencing (RNA-seq)

In terms of RNA-seq, total RNA was extracted from HUVECs using the Trizol reagent (Invitrogen, CA, USA). The integrity of RNA samples was evaluated using the agarose gel electrophoresis and the Agilent 2100 Bioanalyzer (Agilent Technologies). Subsequently, 2× 150 bp paired-end sequencing (PE150) was carried out on an Illumina Novaseq™ 6000 (Gene Denovo Technology Co., Ltd., China) according to the manufacturer’s protocol. For bioinformatics analysis, RNA-seq data (n = 3 per group) were normalized using DESeq2 median-of-ratios method with PCA-based batch correction, and differentially expressed (DEGs) were identified at adjusted p-adjust < 0.05 and |log2(Fold Change)|> 1 with Benjamini–Hochberg FDR genes correction. Functional enrichment analysis of upregulated genes in the CM (Sal A) group was performed for both Gene Ontology (GO) terms and KEGG pathways which were carried out by topGO and clusterProfiler.

### Immunofluorescence staining

Cell immunofluorescence was performed as previously described with minor modifications [20]. In brief, HUVECs were fixed with 4% paraformaldehyde (PFA), permeabilized with 0.1% Triton X-100 and then blocked with 5% bovine serum albumin (BSA) solution. The HUVECs were then incubated with indicated primary antibodies, followed by incubation with the corresponding fluorescent secondary antibodies. The nuclei were counterstained with DAPI and the immunofluorescence cell images were acquired by a Leica SP8 confocal microscopy (Leica SP8 STED 3X, Heidelberg, Germany).

In terms of the tissue immunofluorescence staining, whole-mount immunofluorescence staining of mouse retina was conducted as previously described with minor modifications [21]. Briefly, the eyes were carefully collected from STZ-induced diabetic mice, and then the harvested eyes were fixed in 4% PFA solution in phosphate-buffered saline (PBS) for 2 h on ice. For the dissection of retinas, the cornea, lens, sclera, and hyaloid vessels were carefully removed. The retinas were permeabilized and blocked in PBS containing 0.5% Triton and 5% BSA overnight at 4 °C, followed by incubation with indicated primary antibodies diluted at a ratio of 1:100 in PBS containing 0.1% Triton X-100 and 1% BSA overnight at 4°C. Subsequently, the retinas were washed in PBS and then incubated with the corresponding fluorescent secondary antibodies overnight in the dark at 4°C. After incubation, the retinas were washed five times in PBS, and then flattened and mounted through making four incisions in the fluorescent mounting medium. The images were acquired with the same exposure and gain settings using a two-photon confocal microscope (Leica STELLARIS 8 DIVE, Heidelberg, Germany) or THUNDER Imager (Leica THUNDER Imaging Systems, Heidelberg, Germany). The images of retinal blood vessels with different immunofluorescence staining were analyzed using ImageJ software (National Institutes of Health, USA).

### Evans blue leakage assay

In brief, Evans Blue dye (45 mg/kg) was intravenously administered into the STZ mice and allowed to circulate for 30 min. After the eyes were dissected, one eye from each mouse was fixed with 4% PFA for further fluorescent detection of Evans blue dye using a fluorescence microscope (Leica THUNDER Imaging Systems, Heidelberg, Germany), and the other retinas were treated with formamide overnight at 70  C to extract the Evans blue dye. The absorbance of the extracts at 620 nm was measured using a microplate reader (EnSpire PerkinElmer, Waltham, USA).

### Tube formation assay

The tube formation capability of HUVECs was examined by virtue of the in *vitro* tube formation assay. Briefly, the Matrigel was plated into a 96-well plate and incubated at 37 ℃ for 20 min to allow polymerization. Subsequently, 4× 10 [4] HUVECs were cultured on the top of Matrigel layer, and the images of formed capillary-like structures were captured under an inverted optical microscope (Zeiss Axio vert A1, Oberkochen, Germany).

### Wound migration assays using the ibidi culture-inserts

HUVECs and SVG p12 cells were resuspended at the concentration of 3 × 105 cells/ml, and 70 µl of each suspension was loaded into separate chambers of an ibidi Culture-Insert 2 Well (Ibidi, Germany). After 24 h, the insert was gently removed with sterile tweezers, and 2 ml of cell-free medium was added. Cell migration was imaged by a THUNDER Imager (Leica THUNDER Imaging Systems, Heidelberg, Germany).

### Endothelial barrier integrity assay

Commercialized organ chip kit (SynVivo, AL, USA) were used to prepare three dimensional (3D) models to mimic the crosstalk between ECs and astrocytes. Each chip consists of a top lane used to culture ECs to form endothelial barrier, a middle lane filled with an extracellular matrix (ECM) gel and a bottom lane plated with astrocytes. 2 days after cell seeding, the vascular compartment was replenished with fresh EC medium supplemented with FITC-dextran (YEASEN, Shanghai, China). The plate was then imaged using a THUNDER Imager (Leica THUNDER Imaging Systems, Heidelberg, Germany) at different times (0 and 40 min). Fluorescence intensity representing FITC-dextran migrating from the vascular compartment to astrocytes was quantified using ImageJ software and apparent permeability (P app) values were calculated.

### Spheroid sprouting assay

The 3D EC spheroids were generated using the hanging drop method as previously described [22]. After 24 h, the spheroids were harvested and embedded in the collagen matrix containing 1:1 20% fetal calf serum (FCS)-methylcellulose and rat-tail type I collagen matrix (Corning, NY, USA) in a low attachment 24-well plate. Spheroid sprouting was induced by supplementing the medium with 25 ng/ml recombinant mouse VEGF-A. Following 8 h of incubation, the 3D EC spheroids were imaged using an inverted microscope (Zeiss Axio vert A1, Oberkochen, Germany), and spheroid sprouting was quantified with ImageJ software.

### HUVEC permeability assay

HUVEC permeability assay was performed as previously described with minor modifications [18]. In brief, 1 × 105 HUVECs in fibronectin-coated 3 µm transwells (Corning, NY, USA) were incubated overnight. After 24 h, FITC-dextran (0.1 mg/ml) was added to the top chamber; 90 min later, fluorescence values (520 nm) of the bottom medium were measured using a PerkinElmer EnSpire Multimode Plate Reader (EnSpire PerkinElmer, Waltham, USA).

### Enzyme-linked immunosorbent assay (ELISA)

The conditioned medium (CM) was collected from the SVG p12 cells following different treatments and then centrifuged to remove cell fragments. The levels of sSema4D in the CM derived from SVG p12 cells were measured using ELISA kits according to the manufacturer’s instructions (Enzyme-linked, Shanghai, China).

### Western bolt analysis

In belief, the proteins were extracted from SVG p12 cells or HUVECs using Radioimmunoprecipitation Assay (RIPA) buffer. The proteins were then separated based on size through gel electrophoresis, and subsequently transferred to polyvinylidene fluoride (PVDF) membranes. Following incubation with specified primary and secondary antibodies, protein detection and blot visualization were carried out according to the standard procedures.

### Real-time PCR

Total RNA was extracted from the cells using the Trizol reagent. Subsequently, the total RNA was reversed transcribed to cDNA by using RT SuperMix (Vazyme, China). The cDNA was amplified with specific primers to examine the expression levels of target mRNA using ChamQTM SYBR qPCR Master Mix (Vazyme, China). The primer sequences used in this study were listed in Supporting Table S2.

### Mass spectrometry (MS)-based drug affinity responsive target stability (DARTS) assay

The DARTS analysis was conducted as previously described with minor modifications [23]. Briefly, SVG p12 cells were lysed with M-PER mammalian protein extraction reagent (Thermo Fisher Scientific, MA, USA) for 30 min. Aliquots of the lysates were incubated with the indicated concentrations of Sal A and gently agitated for 2 h at room temperature to allow adequate target engagement. The mixtures were then digested with pronase at the specified ratios for 30 min, mixed with loading buffer (Beyotime, Shanghai, China), and boiled for 10 min. Proteins were resolved by sodium dodecyl sulfate–polyacrylamide gel electrophoresis (SDS-PAGE) and visualized with Coomassie Brilliant Blue. The protein-containing bands in the gels were excised for in-gel digestion, after which the MS sequencing and data analysis were performed.

### Cellular thermal shift assay (CETSA)

CETSA was carried out as previously described with minor modifications [18]. In brief, the SVG p12 cells in the absence or presence of Sal A treatment were equally divided into 12 parts and heated for 3 min under different temperatures. Subsequently, the protein expression level of Sema4D was determined by western blot analysis.

### Microscale thermophoresis (MST) analysis

The MST analysis was conducted as previously described with minor modifications [24]. Briefly, the RED dyes-labelled Sema4D protein was mixed with different concentrations of Sal A. Subsequently, the mixed samples were loaded into the standard capillaries, and the MST analysis was carried out using the Monolith NT.115 MST instrument (NanoTemper, Munich, Germany). The data were analyzed with the MO. Affinity Analysis 3 software.

### Preparation of Liposome@Sal A

#### Lipid composition and thin film preparation

Hydrogenated soy phosphatidylcholine (HSPC), cholesterol hemisuccinate (CHEMS), and 1,2-distearoyl-sn-glycero-3-phosphoethanolamine-N-[methoxy (polyethylene glycol) −2000] (DSPE-PEG2000) were weighed at 14.17 mg, 4.87 mg, and 4.24 mg, respectively, according to a molar ratio of 62:33:5 (total lipid molar amount approximately 18.9 μmol), and dissolved in dichloromethane:methanol (2:1, v/v). The mixed solution was placed in a round-bottom flask and subjected to rotary evaporation at 50℃, 300 W, and 150 rpm to form a uniform lipid thin film.

#### Hydration and ammonium sulfate internal phase

A 250 mM ammonium sulfate solution (pH 5.5–6.0) was prepared and used for subsequent hydration of the lipid film, serving as the internal aqueous phase for active loading. Ammonium sulfate establishes a transmembrane gradient across the liposomal bilayer, wherein ammonium ions (NH_4_^+^) dissociate into NH_3_ and H^+^. NH_3_ can diffuse across the lipid bilayer, whereas SO_4_^2−^ cannot permeate the membrane. This differential permeability constitutes the key property enabling remote active loading. Two milliliters of 250 mM ammonium sulfate solution were added to the lipid film, and hydration was performed by stirring at 700 rpm at room temperature for 30 min to obtain a liposomal suspension.

#### Sonication and size control

The liposomal suspension was placed in a glass vial in an ice bath and subjected to probe sonication (120 W) for 10–15 min. This step controls particle size (approximately 80–150 nm) via sonication. Subsequent dialysis using a 30K MWCO membrane further removes small liposomal fragments and free molecules.

#### Ammonium gradient establishment

The sonicated liposomal suspension was transferred to a dialysis membrane with a molecular weight cut-off of 30 K and dialyzed overnight against PBS buffer (pH 7.4). The PBS serves to remove external ammonium sulfate, thereby establishing a transmembrane ammonium gradient, while maintaining the physiological pH and osmotic pressure of the external environment to prevent liposomal aggregation or rupture.

#### Drug loading and purification

The above liposomal suspension was co-incubated with Sal A (1.8 mg) for 30 min to allow Sal A to translocate across the membrane into the liposomal interior. The Sal A-loaded liposomes were then dialyzed against PBS for 2 h to remove unencapsulated free Sal A, yielding Liposome@Sal A. The final liposomal suspension was sterilized by passage through a 0.22 μm nylon membrane filter under aseptic conditions.

### Characterization of Liposome@Sal A

The particle size, polydispersity index (PDI), and zeta potential of Liposome@Sal A were conducted by dynamic light scattering (DLS) using Zetasizer Nano-ZS (Malvern Instruments Ltd., U.K) at room temperature. Meanwhile, the morphology of the liposomes was examined by transmission electron microscopy (TEM). The detailed procedure was conducted as follows: small amounts of liposomes were dropped on the 200-mesh copper grid and stained with a 1% (w/v) uranyl acetate solution for 15 min. After drying naturally at 25 ℃, the samples were imaged by JEM-2100 Time-resolved TEM system (JEOL, Japan). Subsequently, ultraviolet spectrophotometry was utilized to determine the encapsulation efficiency and drug loading. Firstly, Sal A was diluted to a series of different concentration gradients in acetonitrile to obtain a standard curve. next, appropriate amounts of the Liposome@Sal A samples were taken for freeze-drying, followed by that appropriate amount of acetonitrile and sonicate were added and incubated for 30 min to ensure that the liposomes were completely ruptured and the encapsulated Sal A was released. Finally, absorbance of Sal A in the sample was measured, and the amounts of Sal A loaded in the liposomes were calculated based on the standard curve. The encapsulation efficiency (EE%) and drug loading (DL%) of Sal A were calculated using the following formulas:

$${\text{EE }}\left( \% \right)\, = \,{\mathrm{L}}.{\text{Sal A}}/{\mathrm{T}}.{\text{Sal A}}\, \times \,{1}00$$

$${\text{DLC }}\left( \% \right)\, = \,{\mathrm{L}}.{\text{Sal A}}/\left( {{\mathrm{T}}.{\mathrm{Lipo}}\, + \,{\mathrm{T}}.{\text{Sal A}}} \right)\, \times \,{1}00$$

Whereas L.Sal A represented the amount of Sal A loaded in the liposomes, T.Sal A indicated the total amount of Sal A initially added, and T.Lipo was the total amount of lipids added (including HSPC, CHEMS, and DSPE-PEG2000).

### Stability test of Liposome@Sal A

Two groups of samples including Liposome and Liposome@Sal A were prepared for stability test, with three independent samples in each group. At the time points of 1, 3, 5, 7, 9, 15, and 30 days, DLS technology was utilized to measure the particle size of the samples. Through observing the changes in particle size over time, the stability of the liposomes was determined.

### Sample preparation for LC–MS/MS analysis

The sample preparation was conducted as previously described with minor modifications [25]. After enucleation at each time point, the eyeball was promptly rinsed thoroughly with PBS, and adherent tissue was carefully removed prior to tissue homogenization. The sample was then homogenized with 10 volumes of acetonitrile (1 mL) using a tissue homogenizer for 3–5 min, followed by ultra-centrifugation at 13,000 rpm for 15 min at 4 °C. The resulting supernatant was dried under a gentle stream of nitrogen, and the residue was reconstituted in 700 μL of 20% acetonitrile. After centrifugation at 13,000 rpm, the supernatant was collected. Finally, 2 μL of the reconstituted solution was injected into the column-switching LC–MS/MS system for analysis. For plasma samples, proteins were precipitated by adding 4 volumes of acetonitrile, followed by vortexing for 3 min. The mixture was then centrifuged at 13,000 rpm, and 600 μL of the supernatant was transferred to a fresh tube and dried at low temperature. The residue was reconstituted in 700 μL of 20% acetonitrile, centrifuged at 13,000 rpm, and the supernatant was collected, filtered through a membrane, and 2 μL was injected into the LC–MS/MS system for analysis.

### Statistical analysis

Unless otherwise indicated, all data were presented as mean ± standard deviation (SD). Statistical analysis was performed using GraphPad Prism software (Version 8.0, San Diego, USA), based on unpaired Student’s test for comparisons between two groups and one-way ANOVA analysis for more than two groups. P values were denoted in figures as: not significant [ns], p > 0.05, ^#^p ≤ 0.05, ^##^p ≤ 0.01, ^###^p ≤ 0.001 (model group vs control group); ^*^p ≤ 0.05, ^**^p ≤ 0.01, ^***^p ≤ 0.001 (Sal A-treated group vs model group).

## Results

### Sal A attenuated pathological retinal vascular dysfunction in STZ-induced DR

Vascular abnormalities, such as acellular capillaries, intraretinal microvascular dysfunction and vascular leakage, are deemed to be typical pathological features of DR. The STZ-induced diabetic mouse model recapitulates key aspects of human DR. Using CD31 and isolectin B4 (IB4) staining, we found a significant increase in acellular capillaries in diabetic retinas compared to non-diabetic controls, suggesting a successful DR induction. Notably, treatment with 10 mg/kg Sal A significantly reduced the formation of acellular capillaries compared to vehicle control (Fig. S1).

**Fig. 1:**
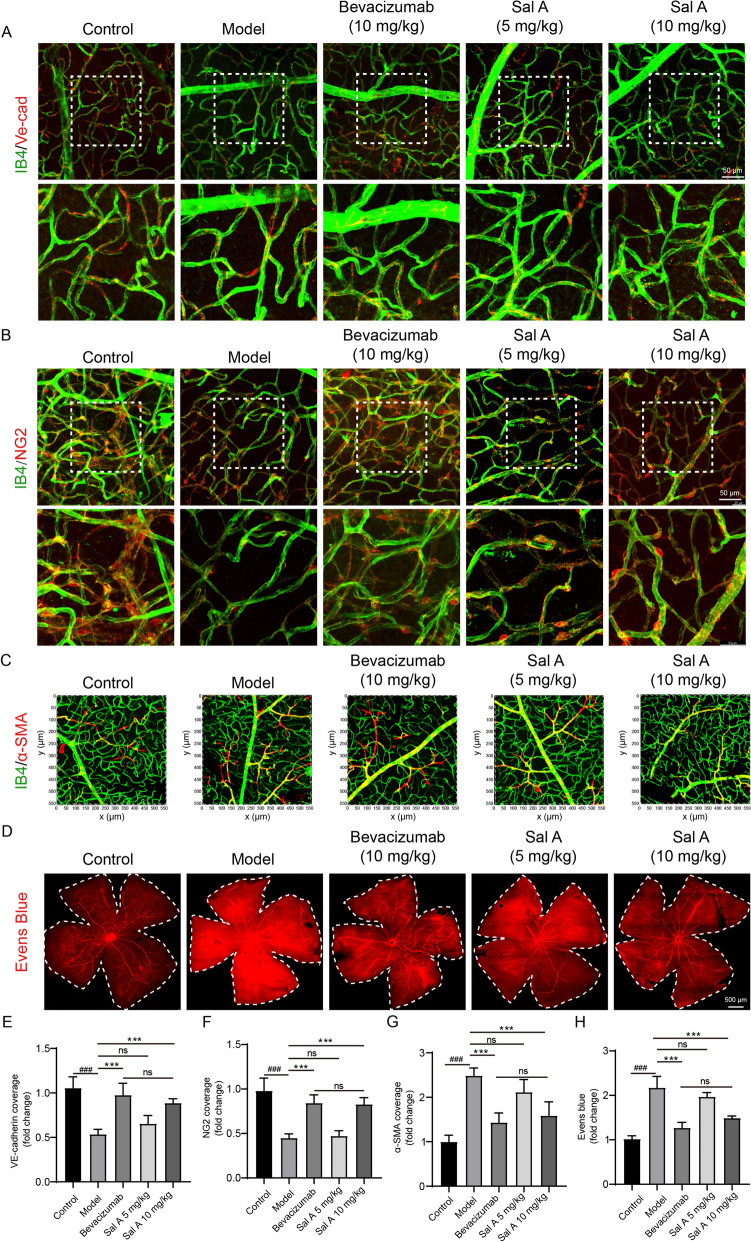
Sal A attenuated pathologic retinal vascular dysfunction in STZ-induced DR. **A** Representative immunofluorescence images of co-staining for IB4 and VE-cadherin (Ve-cad) in the retinas of STZ-induced diabetic mice treated with vehicle control, Bevacizumab and different concentrations of Sal A. The representative regions were highlighted and magnified with white boxes, n = 6. Scale bar: 50 μm. **B** Representative immunofluorescence images of co-staining for IB4 and NG2 in the retinas of STZ-induced diabetic mice treated with vehicle control, Bevacizumab and different concentrations of Sal A. The representative regions were highlighted and magnified with white boxes, n = 6. Scale bar: 50 μm. **C** Representative immunofluorescence images of co-staining for IB4 and a-SMA in the retinas of STZ-induced diabetic mice treated with vehicle control, Bevacizumab and different concentrations of Sal A acquired by two-photon microscopy, n = 6. **D** Evans Blue assay was performed to test the vascular leakage in whole-mount retinas of the STZ-induced diabetic mice treated with vehicle control, Bevacizumab and different concentrations of Sal A, n = 6. Scale bar: 500 μm. **E** Quantification of relative VE-cadherin expression in the retinas for (A), n = 6. **F** Quantification of relative NG2 expression for (B), n = 6. **G** Quantification of relative a-SMA expression for (**C**), n = 6. **H** The absorbance values of extracted Evans Blue dye in the retinas of STZ-induced diabetic mice treated with vehicle control, Bevacizumab and different concentrations of Sal A, n = 6. Data are presented as Mean ± SD. ^##^*P* < 0.01, ^###^*P* < 0.001 vs. Control group; ^***^P < 0.001 vs. Model group; ns represents not significant

To further assess vascular integrity, we stained retinal flat mounts for vascular-endothelial-specific cadherin (VE-cadherin), a crucial regulator of endothelial barrier integrity. Interestingly, in diabetic retinas, VE-cadherin staining was discontinuous compared with controls, indicating a disruption in vascular integrity. In contrast, 10 mg/kg Sal A-treated diabetic mice displayed significantly improved VE-cadherin continuity in microvessels, comparable to Bevacizumab, a well-known drug used to treat DR in the clinic (Fig. 1A and 1E). Furthermore, we also examined the coverage of pericytes on blood vessels via staining by IB4 with cneural/glial antigen2 (NG2). In diabetic retinas, pericyte coverage, as indicated by NG2 positive staining, was decreased by more than 50% compared to control retinas (Fig. 1B and 1 F). Nevertheless, both Sal A (10 mg/kg) and Bevacizumab significantly restored NG2-positive staining in the diabetic retinas, suggesting restoration of pericyte-endothelial interactions and improved pericyte coverage. In addition, it has been widely held that a-smooth muscle actin (a-SMA) is an important marker for capillary matrix remodeling during pathological angiogenesis, which is abundantly expressed in myofibroblasts [26]. The expression of a-SMA has also been validated to be increased in DR [27]. Interestingly, Sal A treatment led to a marked reduction of α-SMA expression (Fig. 1C and 1G), indicating that Sal A is able to retard the capillary matrix remodeling.

Pericyte loss, acellular capillary formation and capillary matrix remodeling contribute to retinal vascular integrity defects, leading to striking vascular leakage [26]. To further determine the therapeutic effect of Sal A in early DR, Evans Blue leakage assay was performed to assess retinal vascular barrier function in the diabetic mice. As shown in Fig. 1D, the fluorescence intensity of Evans Blue dye in the retinal vessels was significantly higher in diabetic mice than that in control mice, implying elevated vascular leakage. Notably, intravitreal injection of 10 mg/kg Sal A significantly diminished the fluorescence intensity of Evans Blue dye compared to vehicle control (Fig. 1H), demonstrating improved vascular barrier integrity. Collectively, these findings indicate that Sal A effectively restores vascular structural and functional integrity in the diabetic retinas, with therapeutic effects comparable to Bevacizumab.

### Sal A reversed the hyperactivated phenotypes of ECs mediated through astrocytes

**Fig. 2:**
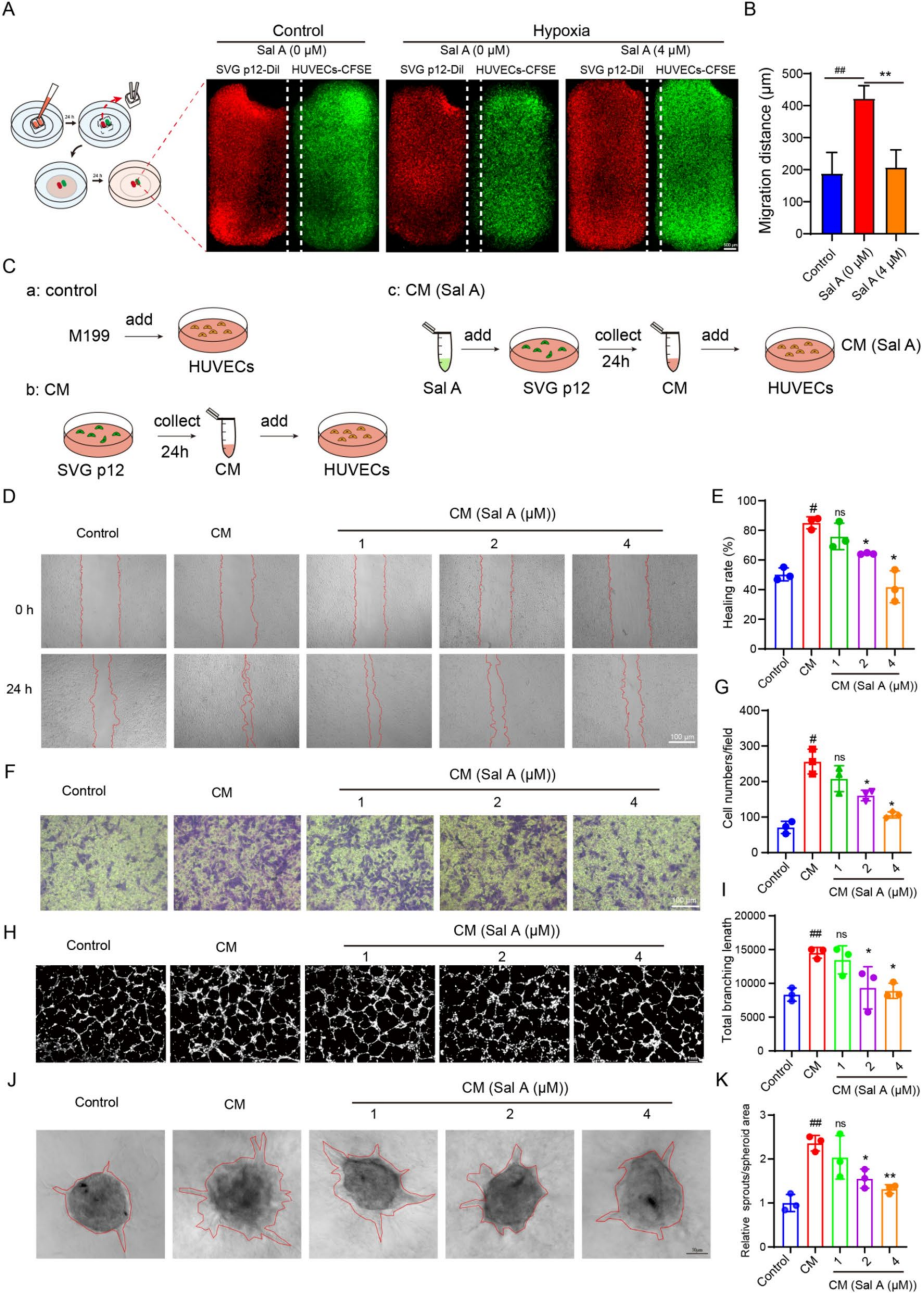
Sal A reversed the hyperactivated phenotypes of ECs mediated through astrocytes. **A** Left: schematic diagram of migration of ECs towards astrocytes by virtue of IBIDI culture-insert 2 well system. Right: Representative fluorescence images of HUVECs migrating towards SVG p12. Scale bar: 500 μm. **B** Quantification of the distance of HUVECs migrating towards SVG p12, n = 3. **C** Schematic diagram of the CM-associated experiments. **D**–**E** The horizontal migration of HUVECs was detected by the wound healing assay, n = 3. Scale bar: 100 μm. **F**–**G** The vertical migration of HUVECs was detected by the transwell migration assay, n = 3. Scale bar: 100 μm. **H** Representative images of tube formation of HUVECs in different groups. Scale bar: 1 mm. **I** Quantification of the total tube length, n = 3. **J** Representative images of sprouting of EC spheroids. Scale bar: 50 μm. **K** Quantification of the sprout-to-spheroid area ratio in the EC spheroids, n = 3. Data are presented as Mean ± SD. ^#^*P* < 0.05, ^##^*P* < 0.01 vs. Control group; ^*^P < 0.05, ^**^P < 0.01 vs. CM group; ns represents not significant

It has been increasingly recognized that astrocytes serve as a critical contributor to result in damaged endothelial function in DR [28] and hypoxia has long been regarded as a hallmark of retinal vascular injury in diabetes mellitus, we further investigated whether Sal A could influence the interactions between astrocytes and ECs under hypoxic conditions. Intriguingly, astrocytes were inclined to remarkably augment the migration distance of ECs under hypoxic conditions by virtue of the ibidi Culture-Insert 2 well system (Fig. 2A and 2B). Of note, the increased migration of ECs induced by astrocytes was prominently reversed by Sal A at the concentration of 4 μM, a concentration that failed to affect cell viability (Fig. S2A and S2B). In this regard, this non-contact co-culture system indicated that the CM derived from astrocytes might play an important role in modulating the biological behaviors of ECs. Therefore, we subsequently examined the impacts of astrocyte-derived CM on different biological functions of ECs through taking advantage of various in vitro culture systems. More specifically, three types of media were used to stimulate the HUVECs: M199 medium (control), CM from hypoxic astrocytes (SVG p12 cells), and CM from Sal A-pretreated hypoxic astrocytes (CM (Sal A)) (Fig. 2C). Notably, in comparison to the control group, HUVECs exposed to CM from hypoxic astrocytes exhibited prominently augmented migration. However, Sal A was capable of significantly inhibiting the migrations of HUVECs in the CM-treated group, as shown by both the wound healing assay (Fig. 2D and 2E) and transwell migration assay (Fig. 2F and 2G). Meanwhile, the proliferation of endothelial cells among the groups showed no significant difference (Fig. S2C and S2D). Additionally, we evaluated the effects of astrocyte-CM under hypoxic or high-glucose (HG) conditions on endothelial migration and function. The results uncovered that although both Hypoxia-CM and HG-CM promoted endothelial migration and impair barrier function, hypoxia-CM exerted a more pronounced effect on ECs (Fig. S2E-S2H). Consequently, we focused on the hypoxia-induced model in our subsequent studies.

Given that development and sprouting of new capillaries from resident ECs are deemed to be the hallmarks of angiogenesis [29], we then evaluated the angiogenic potential of ECs by virtue of the tube formation and the spheroid sprouting assays. It was demonstrated that the CM derived from Sal A-pretreated hypoxic astrocytes suppressed the tube formation (Fig. 2H and 2I) and angiogenic sprouting (Fig. 2J and 2 K) of HUVECs in a dose-dependent manner as comparison to the CM-treated HUVECs. In order to further explore whether Sal A could exert any direct impacts on the migration of ECs, various concentrations of Sal A were applied to HUVECs. Intriguingly, no significant differences were observed in different biological functions of ECs including proliferation, migration and tube formation between CM and CM derived from Sal A-pretreated hypoxic astrocytes, indicating that Sal A was able to prevent the migration of HUVECs via influencing the interactions between astrocytes and ECs rather than directly affecting the biological functions of ECs (Fig. S2I-S2L). Taken together, our findings illustrate that Sal A tends to restrict the hyperactivated phenotypes of ECs mediated through astrocytes in vitro.

### Sal A rescued the disruption of the endothelial barrier triggered by astrocytes

**Fig. 3:**
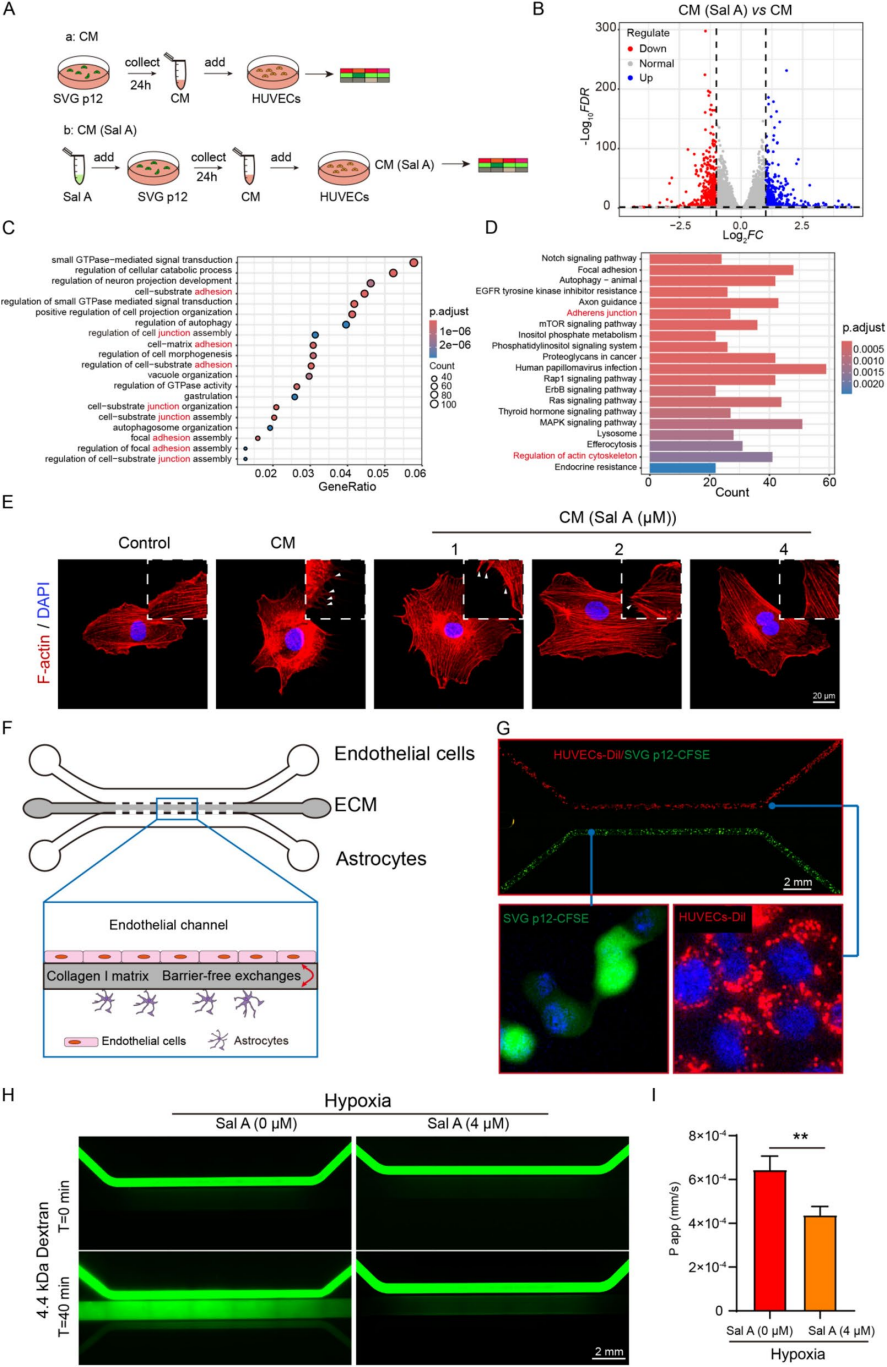
Sal A rescued the disruption of the endothelial barrier triggered by astrocytes. **A** Schematic diagram of the experiment procedures involving preparing RNA-seq samples, n = 3. **B** Volcano plot showing the differentially expressed genes. **C** GO analysis showing the biological processes based on the upregulated genes. **D** KEGG enrichment analysis showing the signaling pathways based on the upregulated genes. **E** Representative immunofluorescence images for F-actin in the HUVECs following different treatments, n = 3. Scale bar: 20 μm. **F** Schematic diagram showing the microfluidic chips and experimental design. **G** Representative images illustrating the spatial distribution of HUVECs (red, right panel) and SVG p12 cells (green, left panel) within the microfluidic chips. Scale bar: 2 mm. **H** Representative images of 4.4 kDa FITC-dextran in chips after a 40 min incubation. Scale bar: 2 mm. **I** Quantification of 4.4 kDa FITC-dextran apparent permeability (P app) values in chips, n = 3

To better gain insight into the molecular mechanisms underlying that Sal A influenced the interactions between astrocytes and ECs, we thus carried out the RNA-seq to examine the transcriptome of HUVECs in the CM and CM (Sal A) groups. Of note, there were a total of 2757 genes that were significantly upregulated and 1267 genes that were dramatically downregulated in response to CM (Sal A) treatment when compared with the CM group (Fig. 3A and 3B). GO analysis of the upregulated genes by the treatment of CM (Sal A) unveiled that the majority of the biological processes were associated with focal adhesion and cell junction (Fig. 3C). Meanwhile, KEGG pathway enrichment analysis unraveled that the upregulated genes were predominantly involved in adherens junctions (AJs) and regulation of actin cytoskeleton (Fig. 3D). To validate the transcriptomic findings, qPCR was performed for key junctional genes (*CDH5*, *CLDN5*, and *TJP1*) in ECs treated with either CM supplemented with Sal A or CM alone. The results showed that junctional gene expression was significantly upregulated in the CM (Sal A) group relative to the CM control, which was consistent with the RNA-seq data (Fig. S3A). It is well known that the formation of F-actin-rich protrusions serves as a classic hallmark of cell motility, facilitating cellular extension, adhesion, and propulsion [30]. Notably, in comparison to the control group, HUVECs exposed to CM from hypoxic astrocytes displayed prominent F-actin-rich protrusions. Nevertheless, CM derived from the Sal A-pretreated hypoxic astrocytes robustly reduced the number of F-actin-rich protrusions in HUVECs compared to CM derived from hypoxic astrocytes (Fig. 3E). Moreover, MYO10 — an important marker of filopodia that cross-links multiple cytoskeletal proteins —was profoundly upregulated in the HUVECs exposed to CM, which could be rescued in response to CM (Sal A) treatment in a dose-dependent manner (Fig. S3D and S3G). To this end, these findings further elucidated the potential mechanisms by which Sal A reversed the astrocyte-driven hyperactivated phenotypes of ECs.

Subsequently, we were inclined to shed light on whether CM (Sal A) would be able to regulate the protein expression levels of AJs-related components (*e.g.*, VE-cadherin) and tight junctions (TJs)-associated molecules (*e.g.*, ZO-1, Claudin5). Intriguingly, it was demonstrated that CM treatment significantly downregulated the expression levels of VE-cadherin, ZO-1 and Claudin5 in the HUVECs, which could be rescued in response to CM (Sal A) treatment in a dose-dependent manner (Fig. S3B-S3F). In fact, endothelial barrier function has been deemed to be closely associated with endothelial junctions. As measured by FITC-dextran leakage and transendothelial electrical resistance (TEER) values in a transwell HUVEC monolayer model, it was shown that CM treatment increased ECs permeability, which could be reversed in the presence of CM (Sal A) treatment dose-dependently (Fig. S3H and S3I). Next, we established a co-culture model of ECs and astrocytes using microfluidic chips. Specifically, 3-lane microfluidic chips were used to enable the co-culture of HUVECs in the top lane to form endothelial barrier and astrocytes in the bottom lane (Fig. 3F and 3G). The barrier integrity of ECs was assessed by calculating P app coefficients based on 4.4 kDa FITC-dextran dyes migrating from vascular compartment to astrocytes. Notably, the treatment with Sal A strikingly attenuated the leakage of 4.4 kDa FITC-dextran dyes through the endothelial barrier (Fig. 3H and 3I). In conclusion, these data suggest that astrocyte-mediated dysfunction of endothelial barrier can be reversed in response to Sal A intervention on the astrocytes.

### Astrocyte-derived sSema4D gave rise to aggressive biological events of ECs

**Fig. 4:**
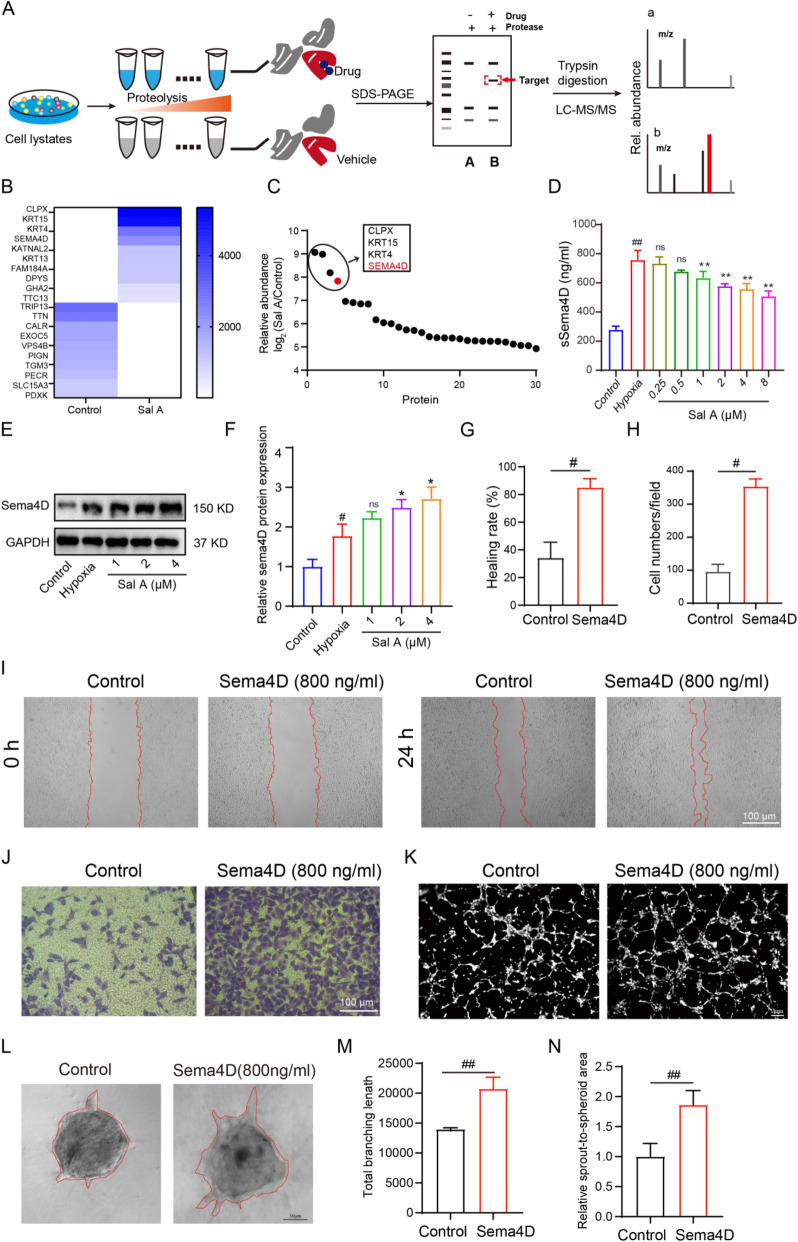
Astrocyte-derived sSema4D gave rise to aggressive biological events of ECs. **A** Schematic diagram of the experiment procedures involving preparing the sample of MS-based DARTS. **B** MS-based DARTS sequencing of associated proteins was performed to identify the potential targets of Sal A. **C** Relative abundance of Sal A target protein candidates. **D** The protein levels of secreted sSema4D in the CM was determined, n = 3. **E**–**F** Western blot analysis for Sema4D in the SVG p12 lysates following different treatments, n = 3. **G** Quantifications of the wound healing rate of HUVECs following different treatments, n = 3. **H** Quantifications of the number of migrated HUVECs following different treatments, n = 3. **I** The migration of HUVECs in the absence or presence of Sema4D was examined by wound healing assay, n = 3. Scale bar: 100 μm. **J** The migration of HUVECs in the absence or presence of Sema4D was examined by transwell system, n = 3. Scale bar: 100 μm. **K** Representative images of formed tube-like structures in the absence or presence of Sema4D, n = 3. Scale bar: 1 mm. **L** Representative images of sprouting of EC spheroids. Scale bar: 50 μm. **M** Quantifications of the total branching length of formed tubes by HUVECs, n = 3. Scale bar: 100 μm. **N** Quantification of the relative sprout-to-spheroid area ratio in the EC spheroids, n = 3. Data are presented as Mean ± SD. ^#^*P* < 0.05, ^##^*P* < 0.01 vs. Control group; ^*^P < 0.05, ^**^P < 0.01 vs. Model group, ns represents not significant

Subsequently, we interrogated the mechanisms underlying Sal A acted on astrocytes to restore endothelial function. The drug affinity responsive target stability (DARTS) assay, regarded as an effective and efficient approach to excavate the direct targets for ligands without additional modifications, was utilized to unlock the potential candidates that are able to interact with Sal A. To this end, we therefore incubated the SVG p12 lysates with different concentrations of Sal A, and observed a Coomassie brilliant blue-stained band at 100–150 kDa where Sal A-treated samples appeared to be more abundant than vehicle control-treated samples (Fig. S4A). Notably, MS analysis unraveled that Clpx, Krt15, Krt4 and Sema4D were the most prominently enriched proteins in the Sal A-treated group relative to that in the vehicle control group (Fig. 4A–4C). Due to that it has been well documented that Sema4D is closely associated with angiogenesis [31], its upregulation suggested that Sal A ameliorated the development of DR at least in part through remodeling the retinal blood vessels by virtue of Sema4D-dependent pathways. Interestingly, it was elucidated that the mRNA level of Sema4D in the astrocytes were significantly higher than that in the ECs under hypoxic condition in vitro (Fig. S4B). More importantly, in the STZ-induced diabetic mouse model, the positive signals of Sema4D were profoundly elevated in the GFAP^+^ glial cells compared to that in the healthy mice as demonstrated by the immunofluorescence staining, indicating that astrocytes serve as a major source of Sema4D, with its expression level strikingly boosted in DR (Fig. S4C). Previous studies have illustrated that Sema4D is prone to be susceptible to proteolytic cleavage to form a soluble variant (sSema4D) that is subsequently released from the cell surface. Of interest, Sal A conspicuously declined the levels of hypoxia-induced sSema4D in the CM derived from astrocytes while dose-dependently augmented the levels of membrane-bound Sema4D on astrocytes (Fig. 4D-4F).

Indeed, it has been widely appreciated that sSema4D has high propensity to exert potent pro-angiogenic and exudative effects in tumors [32]. Nevertheless, its roles in influencing the development of DR remains largely unclear. To further examine the effects of Sema4D on the biological behaviors of ECs in vitro, we therefore performed wound-healing and transwell migration assays. Intriguingly, the results unraveled that the treatment of recombinant Sema4D dramatically enhanced the migration of ECs (Fig. 4G-4J). In addition, tube formation and spheroid sprouting assays delineated that Sema4D was prone to potently promote the formation of tube-like structures and orchestrate angiogenic sprouting, indicative of a pro-angiogenic function of Sema4D (Fig. 4K-4N). To rigorously establish that Sal A regulates endothelial function by suppressing Sema4D shedding from astrocyte membranes, we performed sSema4D rescue experiments and astrocyte-specific Sema4D knockout studies. In Sema4D-knockout astrocytes, hypoxia-induced barrier disruption was abolished, and additional Sal A treatment produced a similar outcome to Sema4D knockout alone. This indicates that the protective effect of Sal A is dependent on astrocytic Sema4D expression. Exogenous sSema4D restored barrier disruption in Sema4D-knockout astrocytes, and Sal A failed to reverse sSema4D-induced barrier dysfunction. This demonstrates that Sal A interacts with membrane-anchored Sema4D on astrocytes, thereby inhibiting its shedding/cleavage into the soluble form, rather than directly neutralizing sSema4D in the extracellular space (Figure S4D-4F). In summary, our data implicate that astrocyte-derived sSema4D drives aggressive biological phenotypes of ECs, and that Sal A counteracted these effects by attenuating sSema4D shedding from the astrocyte membranes.

### Sal A prevented the shedding of sSema4D via directly interacting with the Arg92 residue of Sema4D

**Fig. 5:**
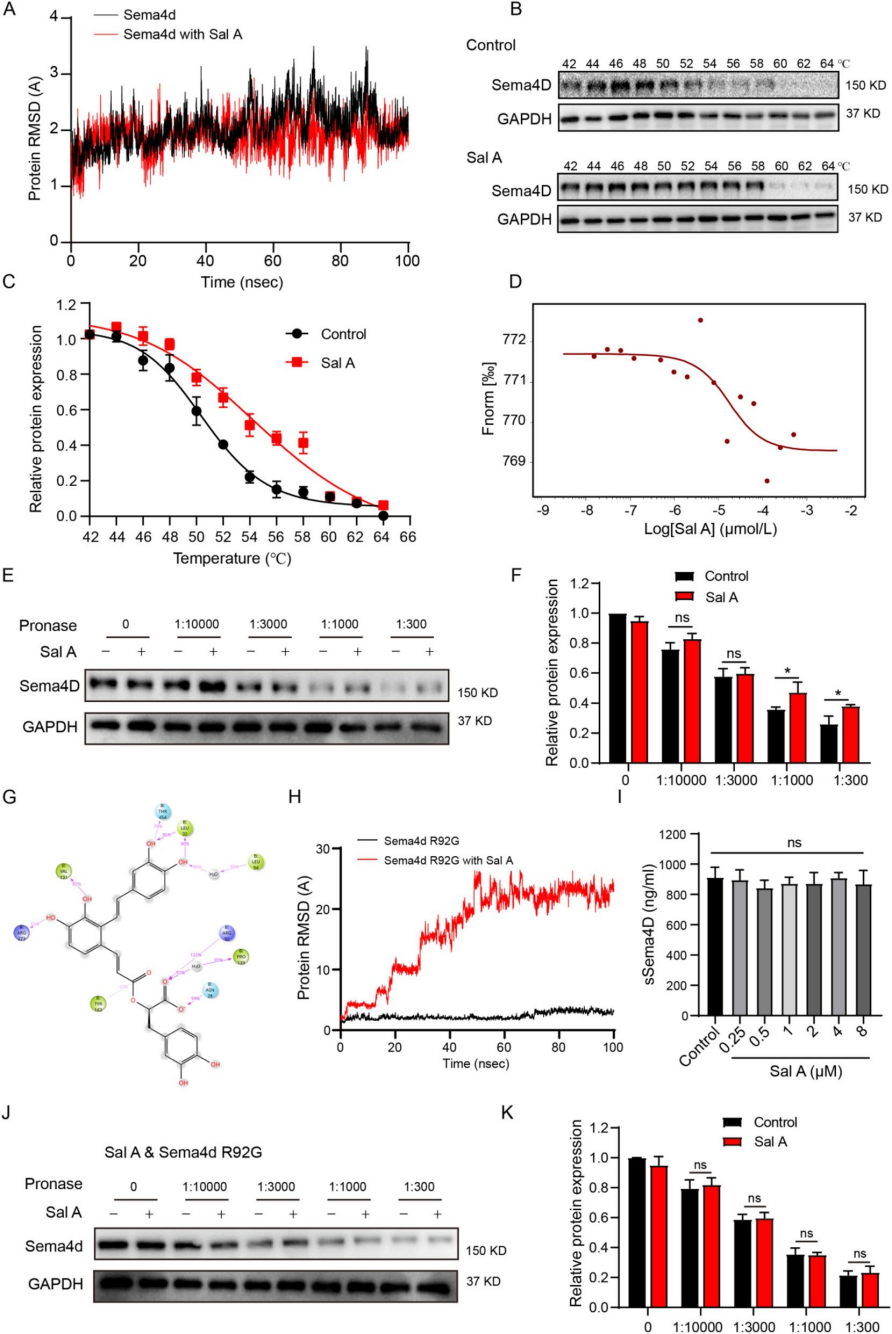
Sal A prevented the shedding of sSema4D via directly interacting with the Arg92 residue of Sema4D. **A** The time evolution of RMSD values for Sema4D protein in the absence or presence of Sal A. **B** The SVG p12 cells were incubated with Sal A, followed by that the cells were collected and subjected to temperature-dependent CESTA assay for Sema4D, representative western blot bands are shown. **C** Quantification of the protein levels for (**B**), n = 3. **D** MST analysis for determining the bind affinity of Sal A to the purified Sema4D protein, n = 3. **E** DARTS assay was performed to determine the binding affinity of Sal A to Sema4D protein, n = 3. **F** The protein expression level of Sema4D was quantified on the basis of densitometric analysis, n = 3. **G** Interaction pattern and probability distribution of binding sites of Sema4D for Sal A. **H** The time evolution of RMSD values for Sema4D protein with R92G mutation in the absence or presence of Sal A. **I** The protein levels of secreted sSema4D in the CM were determined, n = 3. **J** DARTS assay was performed to determine the binding affinity of Sal A to Sema4D protein with R92G mutation, n = 3. **K** The protein expression level of Sema4D was quantified on the basis of densitometric analysis, n = 3. Data are presented as Mean ± SD. *P < 0.05 vs. Control group, ns represents not significant

To further dissect whether there were direct interactions between Sal A and Sema4D, the molecular dynamics simulation assay was performed accordingly. To our surprise, it was elucidated that root mean square deviation (RMSD) of Sema4D in the presence of Sal A exhibited no prominent alterations and remained similar pattern in comparison to that of Sema4D alone, suggesting that Sal A might directly bind to Sema4D (Fig. 5A). Furthermore, we took advantage of the CETSA assay to determine the binding affinity between Sal A and Sema4D. As demonstrated in Fig. 5B and 5 C, the stability of Sema4D protein in the presence of Sal A was much higher under temperature gradients compared to that in the absence of Sal A, further validating that Sal A displayed a direct binding capability to Sema4D. Consistently, MST assay illustrated that Sal A was capable of directly binding to Sema4D protein (Fig. 5D). In line with these findings, DARTS assay pinpointed that there was a reduction in the protease susceptibility of Sema4D in the presence of Sal A than that in the absence of Sal A, indicative of the direct binding of Sal A to Sema4 D (Fig. 5E and 5 F).

Subsequently, molecular dynamics simulations was conducted to predict Sal A’s potential binding mode and binding sites on Sema4D protein, and it was observed that Sal A and Arg92 residue of Sema4D can form a hydrogen-bond interaction with an 122% distribution, suggesting that Arg92 serves as a critical residue of Sema4D to bind with Sal A (Fig. 5G). With the purpose of further clarifying the function of Arg92 residue of Sema4D in maintaining the structural stability of Sal A-Sema4D binding complex, the 100 ns molecular dynamics simulations were performed to exploit the binding capability of Sal A to Sema4D protein with Arg92 residue mutating to Gly92 (Sema4D-R92G mutant protein). Of note, it was shown that mutation of Arg92 residue strikingly altered the RMSD values of the Sal A-Sema4D binding complex (Fig. 5H). Meanwhile, we transfected 293 T cells with the Sema4D-R92G-mutant plasmid and further determined the binding ability of Sal A to the Sema4D-R92G protein by virtue of the DARTS assay. Homoplastically, the binding capability of Sal A to Sema4D protein with R92G mutation was compromised upon the mutation of Arg92 residue (Fig. 5J and 5 K). Notably, Sal A failed to diminish the level of sSema4D in the CM derived from H293T cells (Fig. 5I). In this perspective, these results underscored that Sal A was capable of binding to the Arg92 residue of Sema4D to further reduce the shedding of sSema4D.

### Sema4D disrupted endothelial integrity via leading to the activation of RhoA/ROCK2/pMLC2 signaling cascade

**Fig. 6:**
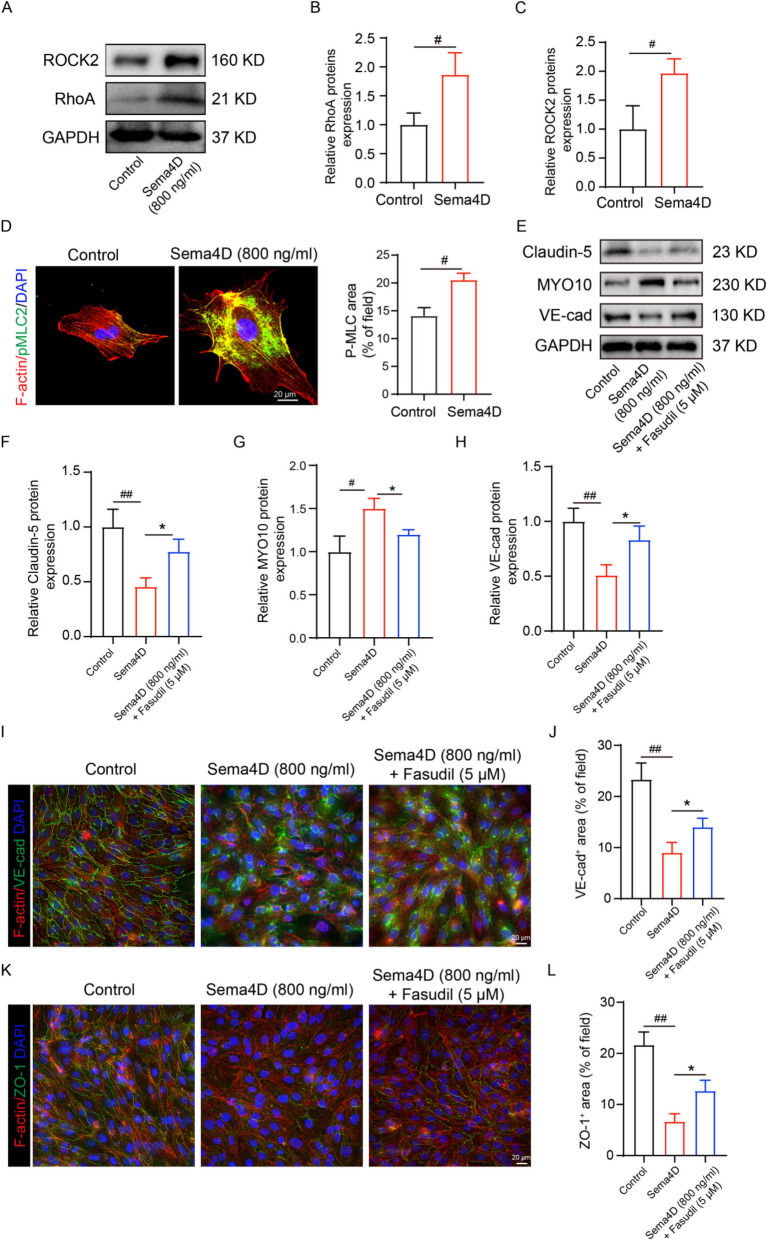
Sema4D disrupted endothelial integrity via leading to the activation of RhoA/ROCK2/pMLC2 signaling cascade. **A**–**C** Western blot analysis for the protein expression levels of ROCK2 and RhoA in the HUVEC lysates following the stimulation of recombinant Sema4D. Representative bands and quantifications are shown, n = 3. **D** Representative immunofluorescence images for pMLC2 in the HUVECs in the absence or presence of Sema4D stimulation, n = 3. Scale bar: 20 μm. **E**–**H** Western blot analysis for Claudin5, MYO10 and VE-cadherin (VE-cad) in the HUVEC lysates following different treatments, n = 3. **I** Representative immunofluorescence images for VE-cad (green)/F-actin (red)/DAPI (blue) in the HUVECs. Scale bar: 20 μm. **J** Quantification of the percentage of VE-cad positive area in the visual field in the HUVECs upon different treatments, n = 3. **K** Representative immunofluorescence images for ZO-1 (green)/F-actin (red)/DAPI (blue) in the HUVECs. Scale bar: 20 μm. **L** Quantification of the percentage of ZO-1 positive area in the visual field in the HUVECs upon different treatments, n = 3. Data are presented as Mean ± SD. ^#^*P* < 0.05, ^##^*P* < 0.01 vs. Control group; ^*^P < 0.05 vs. Sema4D group, ns represents not significant

Subsequently, we intended to analyze the molecular mechanisms by which Sema4D exerted striking effects on disrupting the endothelial integrity. Notably, it has been well documented that RhoA/ROCK2 signaling pathway contributes to hyperglycemia-induced endothelial dysfunction by virtue of remodeling the cytoskeleton, thereby promoting the migration of ECs and yielding the compromised tight junctions [33]. Of interest, western blot analysis illustrated that the treatment with recombinant Sema4D substantially augmented the protein expression levels of RhoA and ROCK2 in the HUVECs (Fig. 6A-6C). Also, the phosphorylation level of MLC2 that is located at the downstream of RhoA/ROCK2 signaling pathway was dramatically boosted following the stimulation of recombinant Sema4D protein on the basis of immunofluorescence staining (Fig. 6D). Moreover, the treatment with recombinant Sema4D profoundly downregulated the expression levels of VE-cadherin, Claudin5 and ZO-1 in the HUVECs as well as upregulated the expression level of MYO10, which could all be reversed in response to ROCK2 inhibitor fasudil on the basis of western blot analysis and immunofluorescence staining (Fig. 6E-6L). As such, these data implied that Sema4D disrupted endothelial integrity through leading to the activation of RhoA/ROCK2/pMLC2 signaling cascade.

### Sal A strengthened endothelial function by virtue of impeding the activation of sSema4D/PlexinB1/RhoA/ROCK/pMLC2 signaling pathway

**Fig. 7:**
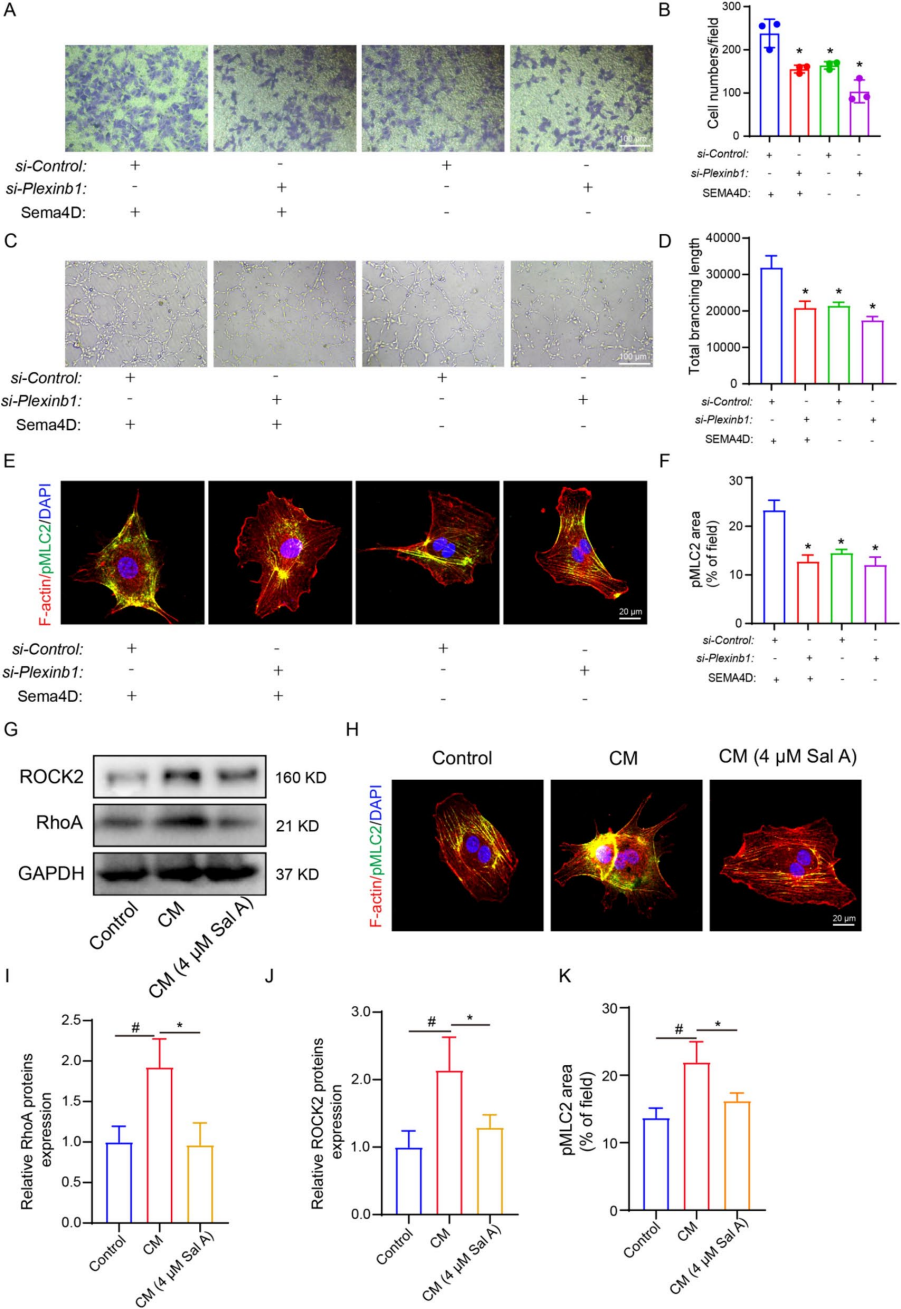
Sal A strengthened endothelial function by virtue of impeding the activation of sSema4D/PlexinB1/RhoA/ROCK/pMLC2 signaling pathway. **A** The migration of HUVECs was examined by the transwell system. Representative images are shown. Scale bar: 100 μm. **B** Quantification of the number of migrated cells, n = 3. **C** Representative images of tube formation of HUVECs following different treatments. Scale bar: 100 μm. **D** Quantifications of the total branching length in the formed tubes by HUVECs, n = 3. **E** Representative immunofluorescence images for pMLC2 in the HUVECs following different treatments. Scale bar: 20 μm. **F** Quantification of the percentage of pMLC2 positive area in the visual field in the HUVECs upon different treatments, n = 3. **G** Western blot analysis for ROCK2 and RhoA in the HUVEC lysates following different treatments. Representative bands are shown, n = 3. **H** Representative immunofluorescence images for pMLC2 in the HUVECs following different treatments, n = 3. Scale bar: 20 μm. **I**–**J** Quantification for the protein expression levels of RhoA and ROCK2 in the HUVEC lysates following the different treatments, n = 3. **K** Quantification of percentage of pMLC2 positive area in the visual field in the HUVECs upon different treatments, n = 3. Data are presented as Mean ± SD. ^#^*P* < 0.05 vs. Control group; ^*^P < 0.05 vs. Sema4D treated group or CM group

We next inspected how Sema4D exerted remarkable effects on influencing the RhoA/ROCK/pMLC signaling pathway in DR. In fact, it has been well accepted that Sema4D is prone to bind with high affinity to its receptor PlexinB1, which ultimately mediates its downstream signaling cascades including RhoA/ROCK/pMLC pathway in ECs [34–36]. In order to parse whether Sema4D could work through PlexinB1 in this context, the expression level of PlexinB1 was silenced in the HUVECs by virtue of a specific siRNA, resulting in fundamental repression of the increased migration capability of ECs stimulated by Sema4D on the basis of tranwell migration assay (Fig. 7A, 7B and Fig. S5A-B). Meanwhile, knockdown of PlexinB1 was also inclined to abolish the Sema4D-elicited elevation in the tube formation ability of HUVECs (Fig. 7C and 7D). Further, immunofluorescence analysis highlighted that silence of PlexinB1 was able to reverse the increased expression level of pMLC2 triggered by Sema4D, suggesting that Sema4D was prone to damage the endothelial function through exerting striking effects on PlexinB1 (Fig. 7E and 7 F). Furthermore, CM (Sal A) was found to rescue CM-mediated enhanced protein expression levels of RhoA, ROCK2 and pMLC2 (Fig. 7G-7K). To confirm that Sal A protects endothelial barrier function through suppression of RhoA/ROCK2, we performed rescue experiments using narciclasine, a potent pharmacological activator of RhoA signaling. Narciclasine (0.1 μM) was applied to HUVECs concurrently with CM (Sal A). The results showed that narciclasine treatment abrogated the pharmacological effects of Sal A on TEER values and endothelial permeability. As a complementary control, co-treatment with the RhoA/ROCK inhibitor fasudil further enhanced Sal A's protective effects on endothelial barrier function, supporting a causal dependency between RhoA/ROCK suppression and endothelial barrier protection (Fig. S5C-D). In this sense, these data deciphered that Sema4D disrupted endothelial function through activating the PlexinB1/RhoA/ROCK/pMLC2 signaling cascade, which can be reversed in the presence of Sal A intervention on the astrocytes.

### Preparation and characterization of liposome@Sal A

**Fig. 8:**
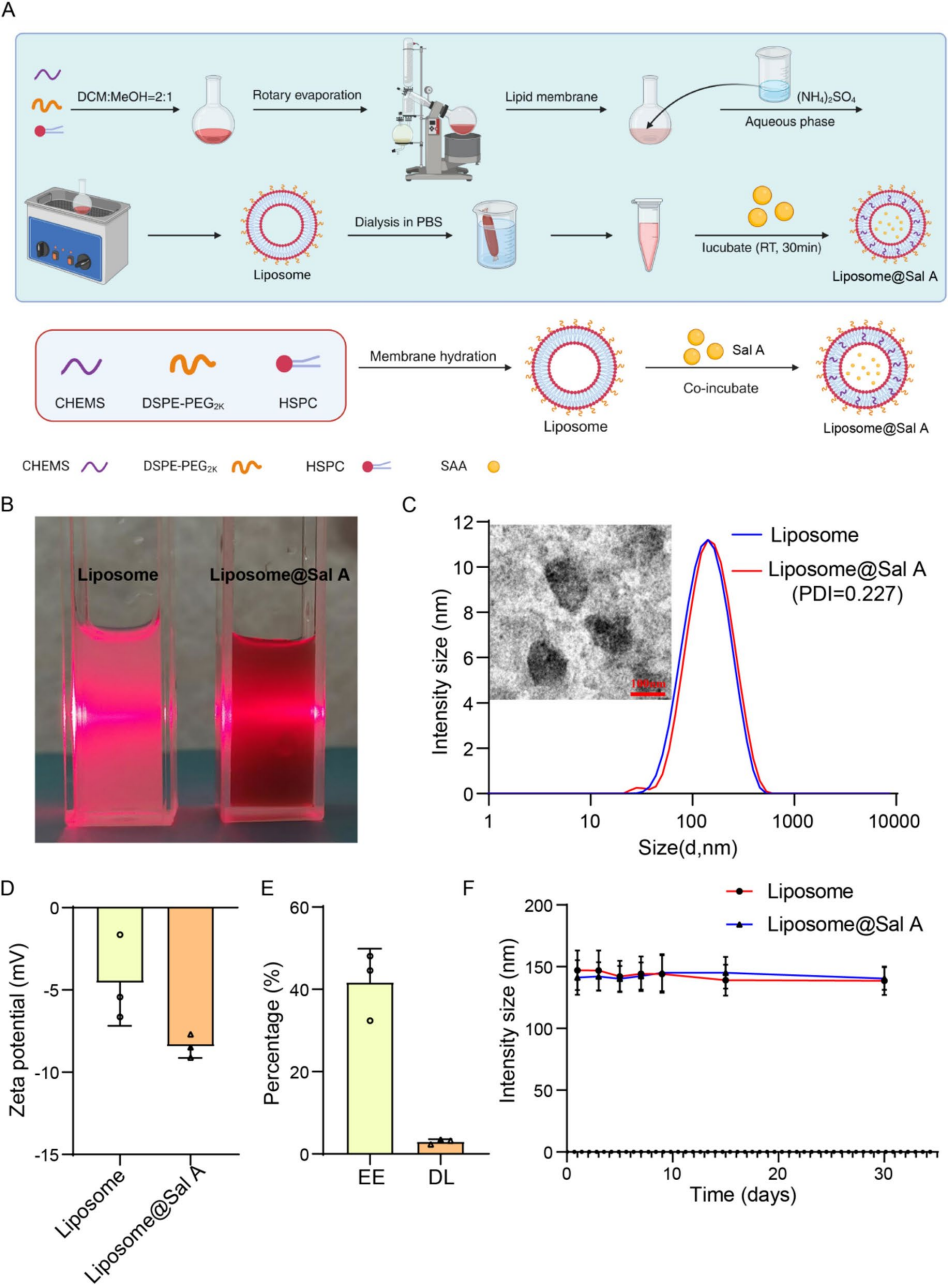
Preparation and characterization of liposome@Sal **A**. **A** Schematic diagram of the structure and synthetic routine of liposome@Sal A. **B** The appearance characteristics of the Liposome@Sal A solution. **C** The topography and particle size of liposome@Sal A. **D** The zeta potential of liposome and liposome@Sal A, n = 3. **E** The EE and DL of liposome@Sal A, n = 3. **F** The stability test of liposome and liposome@Sal A for 30 days, n = 3

Indeed, it has been growing recognized that the application of active ingredients derived from traditional Chinese Medicines is limited due to their defects including low bioavailability. Recently, nanoparticles have been extensively explored as pharmaceutical delivery systems owing to their large specific surface area, strong targeting capability, and sustained-release properties [37]. As versatile drug carriers, liposomes are regarded as artificial lipid vesicles consisting of bilayer membranes and inner aqueous cores for the encapsulation of both lipophilic/hydrophobic molecules and hydrophilic molecules/drugs, respectively. To further investigate whether liposome encapsulation could enhance the potency of Sal A, we first prepared Sal A-loaded liposome nanoparticles (liposome@Sal A). Figure 8A illustrated the detailed preparation process, while Fig. 8B demonstrated the Tyndall effect of empty liposome and liposome@Sal A under light beam irradiation (Fig. 8B). Dynamic light scattering (DLS) analysis uncovered that the average diameter of liposome@Sal A was 138 nm, which was consistent with the transmission electron microscopy (TEM) measurements (Fig. 8C). The polydispersity index (PDI) of liposome@Sal A was 0.227, indicating a relatively uniform particle size distribution. In addition, Fig. 8D displayed the zeta potentials of the empty liposomes and liposome@Sal A, showing negative surface charges of −4.57 ± 2.6% and −8.43 ± 0.71%, respectively. The encapsulation efficiency (EE) and drug loading (DL) of liposome@Sal A were 41.66 ± 8.24% and 2.99 ± 0.59%, respectively (Fig. 8E). Furthermore, size distribution analysis confirmed that liposome@Sal A remained stable for at least 30 days (Fig. 8F). Next, we assessed retinal Sal A levels in the 5 mg/kg liposome@Sal A and free Sal A groups using LC–MS. Notably, retinal Sal A concentrations were significantly higher in the liposome@Sal A group than in the free Sal A group at all tested time points, confirming that liposomal encapsulation enhances retinal drug delivery (Fig. S6A-C). Additionally, we evaluated the plasma pharmacokinetic (PK) profiles of liposome@Sal A. The calculated PK parameters are presented as (Fig. S6D-E), revealing that intravenously administered liposome@Sal A exhibits acceptable metabolic stability. Collectively, the above results prove that the engineered nanoliposome achieved intended design criteria. The particle size, Zeta potential, and structure of the prepared nanoliposome possessed the potential to prolong circulation time of liposome@Sal A in vivo, and promoted its accumulation in the diabetic retinas, thereby enhancing the therapeutic efficacy.

### Liposome@Sal A significantly alleviated the vascular dysfunction in the STZ-induced DR

In order to evaluate the therapeutic effect of Liposome@Sal A in diabetic retinas, we therefore performed electrical impedance analysis on HUVECs in vitro. We found that astrocyte-CM substantially disrupted the stability of HUVEC layer, as evidenced by the decreased TEER value. However, this damaged endothelial barrier was reversed when HUVECS were exposed to the CM from astrocytes preincubated with Liposome@Sal A, suggesting that Liposome@Sal A was able to improve the stability of endothelial barrier, similar to the effects observed in the Sal A-treated group (Fig. S7A). Next, we examined the secreted protein levels of sSema4D in the CM. As depicted in Fig. S7B, Liposome@Sal A markedly reduced hypoxia-induced secretion of sSema4D by the astrocytes, indicating that Liposome@Sal A inhibited the shedding of Sema4D and thereby restored the endothelial function.

**Fig. 9:**
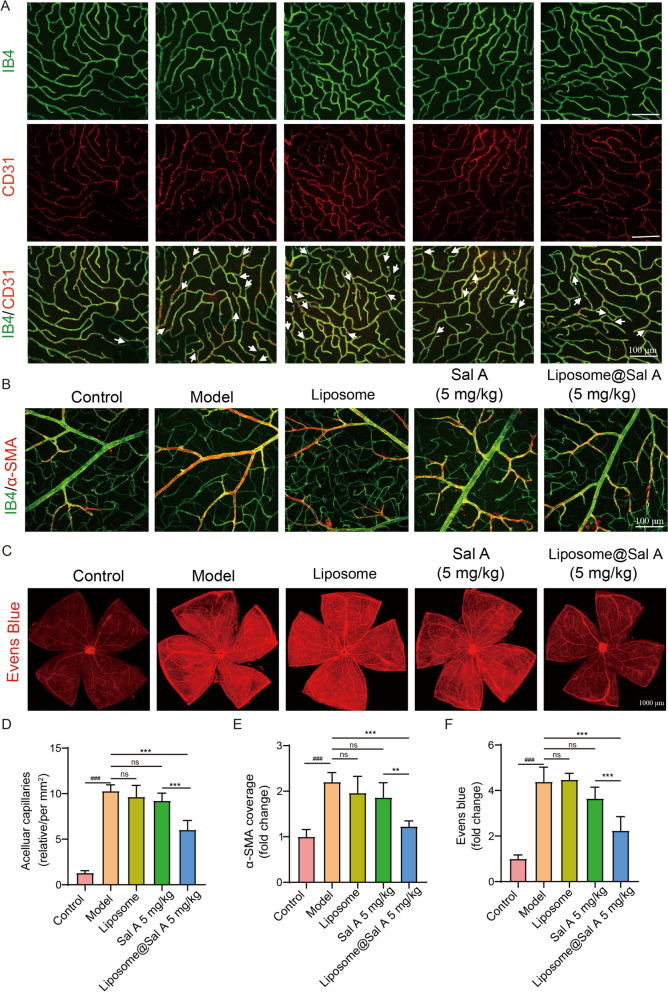
Liposome@Sal A alleviated pathologic retinal vascular dysfunction in vivo. **A** Representative immunofluorescence images of co-staining for IB4 and CD31 in the STZ-induced diabetic mice treated with vehicle control, liposome, Sal A or liposome@Sal A. Arrows indicate the regions of acellular capillaries. Scale bar: 100 μm. **B** Representative immunofluorescence images of co-staining for IB4 and a-SMA in the STZ-induced diabetic mice treated with vehicle control, liposome, Sal A or liposome@Sal A. Scale bar: 100 μm. **C** Evans Blue leakage assay was used to test vascular leakage in whole-mount retinas in the STZ-induced diabetic mice treated with vehicle control, liposome, Sal A or liposome@Sal A. Scale bar: 1000 μm. **D** Quantification of the acellular capillary formation for **A**, n = 6. **E** Quantification of the a-SMA expression for **B**, n = 6. **F** The calculated values of extracted Evans Blue dye were used for quantification for **C**, n = 6. Data are presented as Mean ± SD. ^###^*P* < 0.001 vs. Control group; ^**^P < 0.05, ^***^P < 0.001 vs. Model group, ns represents not significant

Furthermore, we took advantage the STZ-induced diabetic mouse model to investigate the therapeutic efficacy of Liposome@Sal A in vivo. Notably, intravenous injection of 5 mg/kg Liposome@Sal A significantly reduced the number of acellular capillaries and diminished the positive area of a-SMA compared to the treatment of vehicle control in the model group (Fig. 9A, 9B, 9D and 9E). Additionally, 5 mg/kg Liposome@Sal A treatment significantly reduced the fluorescence intensity of Evans Blue dye, indicative of improved vascular integrity (Fig. 9C and 9 F). In contrast, 5 mg/kg of Sal A treatment alone had no significant effects on the number of acellular capillaries, α-SMA^+^ cell coverage, or vascular permeability, demonstrating that liposomal encapsulation improves drug potency via prolonging drug circulation time and increasing drug accumulation at the site of injury. Overall, these data indicated that Liposome@Sal A could enhance the potency of Sal A in restoring vascular structure and function in the diabetic retinas. Given the widespread clinical use of liposomes, Liposome@Sal A has strong potential for clinical translation.

## Discussion

Sal A, one of the predominant water-soluble ingredients extracted from *Salvia miltiorrhiza* Burge, has been documented to exert protective functions against diabetic complications in multiple mouse models, including preventing hepatic fibrosis, mitigating peripheral nerve injury and inflammation, ameliorating early-stage development of atherosclerosis as well as protecting against impaired vascular responsiveness [17, 38–40]. Nevertheless, whether Sal A was able to play any roles in regulating vascular structure and function to influence the progression of diabetic retinas still remained unclarified. In the present study, our data supported the notion that Sal A corrected abnormal retinal vascular structure and function in the STZ-induced diabetic mouse model through intervening in the communications between astrocytes and ECs, demonstrating a robust therapeutic effect comparable to that of Bevacizumab. Consequently, our studies highlighted the therapeutic promise of targeting the interactions between astrocytes and ECs to pave the way for the treatment of DR, in particular for the individuals who fail to respond well to anti-VEGF therapies.

It has been widely appreciated that DR is a diabetic microvascular complication characterized by prominent morphological alterations including thickening of basement membrane and disruption of tight junctions in ECs as well as loss of pericytes, which results in elevated vascular permeability or impaired vascular function. In this study, the diabetic mouse model was successfully established through intraperitoneal administration of STZ, which harbors the advantages of easy operation, high success rate, as well as good repeatability and has been widely employed in both domestic and international studies [41]. In this study, it was found that the treatment of Sal A remarkably alleviated STZ-induced retinal vascular alterations in mice. This was substantiated by prominent increase in the positive regions of VE-cadherin, NG2, and a significant reduction in the positive area of α-SMA. In fact, how acquisition of the contractile protein α-SMA by pericytes results in the aggravated defects of STZ-induced diabetic retinas remains to be elucidated. In brain ischemia models, it has been demonstrated that ischemic pericytes tend to restrict cerebral blood flow (CBF) through constricting capillaries, thereby contributing to no-reflow [42, 43]. To this end, we believe that DR may be closely associated with the abnormalities in pericyte positioning and changes in pericyte contractile property. In addition, the Evans Blue leakage assay illustrated that Sal A ameliorated vascular permeability in the diabetic retinas. In summary, our findings highlighted that Sal A played a fundamental role in correcting the abnormal vascular structure and function in the diabetic retinas.

Notably, in physiological conditions, astrocytes have been validated to rigorously influence endothelial properties, thereby reinforcing blood-retinal barrier (BRB). However, under pathological circumstance, the compelling interactions between astrocytes and ECs exert striking impacts on disrupting barrier functions [28, 44]. Nonetheless, the mechanisms underlying the interactions between astrocytes and ECs are involved in the progression of DR have not been well clarified. In the present study, the ibidi Culture-Insert 2 well co-culture system and the 3D microfluidic multi-cell co-culture model were employed to explore the crosstalk between astrocytes and ECs accordingly. Intriguingly, our data uncovered that the hyperactivated phenotypes of ECs were substantially elevated after being co-cultured with astrocytes, which was found to be reversed in response to Sal A treatment. More interestingly, we demonstrated that Sal A was capable of restoring the impaired endothelial barrier integrity that was ruined following co-culture with astrocytes.

Subsequently, we found that Sal A could bind to Sema4D on astrocytes via the DARTS-MS assay. Of note, the results of molecular dynamics simulations, CETSA, MST and DARTS manifested that Sal A was able to directly bind to the Arg92 residue of Sema4D protein. More interestingly, SalA dose-dependently diminished the hypoxia-induced accumulation of sSema4D in the CM derived from astrocytes in an Arg92-dependent manner. We postulated that by occupying functional pockets and the vicinity of the protease cleavage site in the extracellular domain, Sal A may induce steric hindrance and conformational constraints, thereby potentially inhibiting ADAM10/17-mediated ectodomain shedding of Sema4D [12, 45], although the underlying mechanisms remain to be fully elucidated. It has been well established that sSema4D exert pivotal effects on various conditions including colorectal and lung cancer, as well as ischemic stroke, where it is prone to potentiate angiogenesis and precipitate vascular leakage [46, 47]. Our results deciphered that sSema4D released from the cell membrane of astrocytes profoundly enhanced the migration and angiogenesis of ECs in the diabetic retinas. Moreover, in contrast to VEGF that plays an important role in maintaining normal retinal vascular homeostasis, Sema4D appears to be not essential for the development of retinal blood vessels because Sema4D knockout (KO) mice displays no pronounced changes in light of retinal vascular development compared to wildtype (WT) controls. All of these data indicate that anti-Sema4D possesses merits over anti-VEGF in terms of attenuated non-desirable effects [12].

It has been elaborated that Sema4D is able to bind with a high affinity to its receptor PlexinB1, which further mediates the afferent Sema4D signaling. It has been reported that Sema4D/PlexinB1 signaling cascade regulates endothelial function by virtue of influencing mDIA1-Src signaling [12]. In fact, AJs and TJs of ECs are critical for stabilizing vascular integrity, involving complicated interactions with the actin cytoskeleton [48]. In the present study, we pinpointed that Sema4D/PlexinB1 signaling was capable of yielding endothelial dysfunction as evidenced by decreased VE‐cadherin expression via regulating the RhoA/ROCK/pMLC2 signaling pathway, which was observed to be reversed following the treatment of Sal A. It is conceivable that Sema4D may also provoke dysfunction in VE-cadherin or other junctions-associated proteins in ECs through modulating mDIA1 in the STZ-induced diabetic mouse model, warranting further investigation.

Liposomes, known as a specific type of nanocarrier, have been widely and effectively employed in clinical settings with divergent formulations. In comparison to other nano-delivery systems, liposomes can provide multiple benefits including high biocompatibility, versatility in drug loading, and prolonged circulation time, enabling them to be a conspicuously promising nano-delivery system for numerous biomedical applications [49]. Our study underscored that the therapeutic efficacy of Liposome@Sal A surpassed that of Sal A treatment alone, implying that liposomes can improve the effectiveness via prolonging drug circulation and facilitating drug accumulation at the injury sites.

## Conclusion

**Fig. 10:**
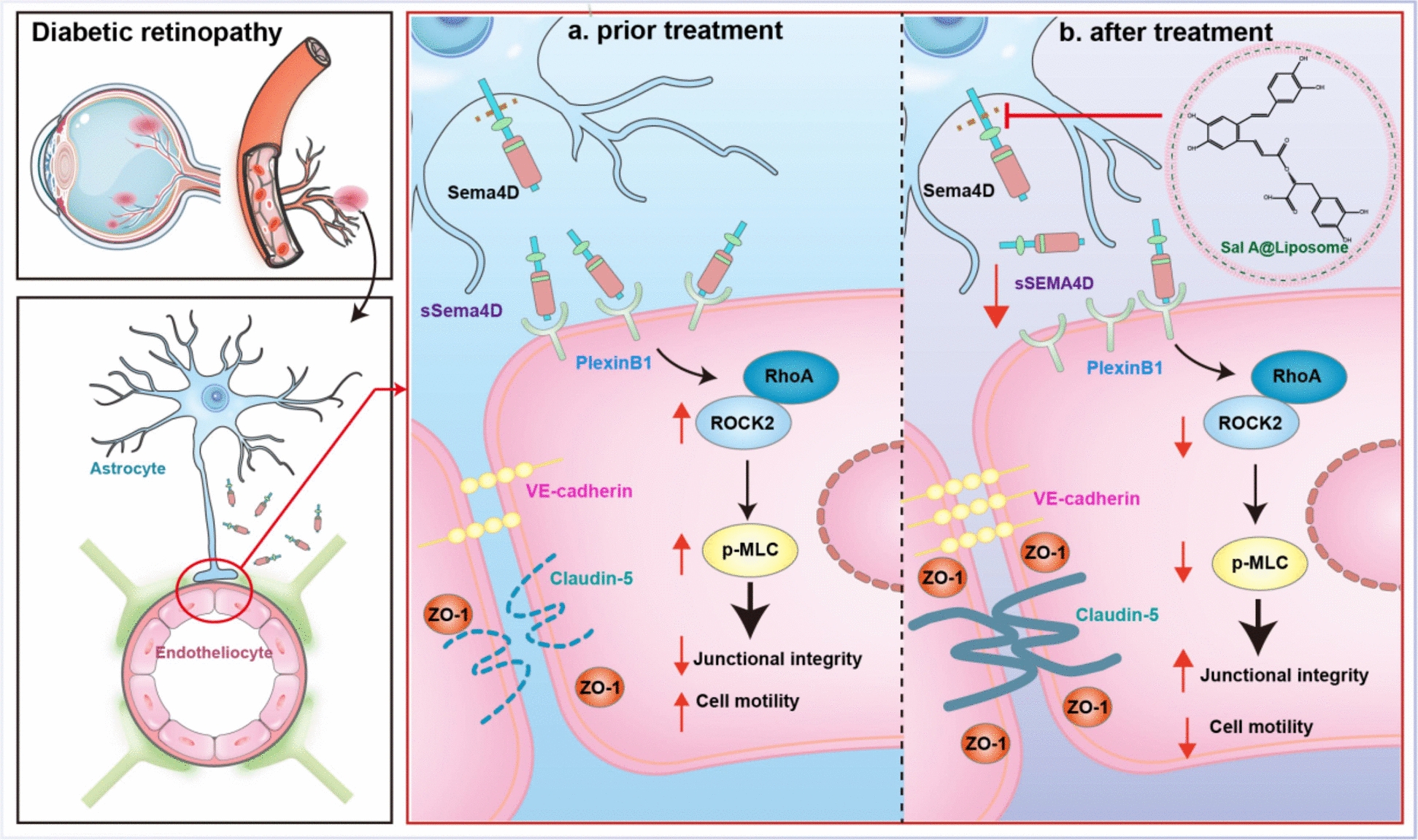
Schematic diagram of Sal A inhibiting the progression of DR. Sal A alters the interactions between astrocytes and ECs via binding to Sema4D on the membranes of astrocytes and determines the fate of endothelial tight junctions by virtue of regulating sSema4D/PlexinB1/RhoA/ROCK/pMLC2 signaling cascade, thereby inhibiting the progression of DR

In summary, our study unveils that Sal A is able to exert striking effects on retarding the progression of DR, which is closely associated with the interactions between astrocytes and ECs. Sal A prevents the shedding of Sema4D by directly binding to Sema4D that is located on the membranes of astrocytes, thereby maintaining the endothelial function by virtue of inhibition of the sSema4D/PlexinB1/RhoA/ROCK/pMLC2 signaling cascade. Moreover, leveraging advancements in nanomedicine, the combination of Sal A with a liposome drug delivery system significantly enhances the therapeutic effectiveness of Sal A in combatting the development of DR. Collectively, our findings delineate that Sal A tends to emerge as a promising drug candidate for improving vascular structure and function in DR (Fig. 10).

## Supplementary Information

Additional file 1

## Data Availability

No datasets were generated or analysed during the current study.

## Abbreviations

SEMA4D: Semaphorin 4D
pMLC2: Phospho-Myosin light chain 2
RhoA: Ras homolog family member A
ZO-1: Zonula occludens protein 1
VE-cad: VE-Cadherin
GAPDH: Glyceraldehyde-3-Phosphate dehydrogenase
GFAP: Glial fibrillary acidic protein
CD31: Platelet endothelial cell adhesion molecule-1
IB4: Isolectin B4
SAA: Salvianolic acid A
FBS: Fetal bovine serum
PBS: Phosphate buffered saline
BSA: Bovine serum albumin
DMSO: Dimethyl sulfoxide
PMSF: Phenylmethanesulfonyl fluoride
KEGG: Kyoto Encyclopedia of Genes and Genomes
GO: Gene Ontology
