# Supplementary material for: Pharmacological manipulation of Sema4D by salvianolic acid A mitigates diabetic retinopathy via inhibiting PlexinB1/RhoA/ROCK/pMLC2 signaling cascade involved in endothelial dysfunction
Source: Chin Med. 2026 Jul 20;21:198. doi: 10.1186/s13020-026-01468-z (PMC13383426; doi:10.1186/s13020-026-01468-z)
Supplement: Supplementary file 1 — Additional file 1 [file 13020_2026_1468_MOESM1_ESM.docx]

**Pharmacological manipulation of Sema4D by Salvianolic acid A mitigates diabetic retinopathy via inhibiting PlexinB1/RhoA/ROCK/pMLC2 signaling cascade involved in endothelial dysfunction**

Weiwei Zheng^1,2,†^, Ling Ning^1,†^, Peiliang Shen^1,2,†^, Jing Ma^1,2^, Chang Yu^1^, Ruiqin Jia^3^, Liwenyu Chen^1,2^, Wei Zou^1^, Yuhua Xu^4^, Yanhong Pan^1,2^, Zhonghong Wei^1^, Qiuhong Shen^1,2^, Chongjin Zhong^1,2^, Aiyun Wang^1^, Wenxing Chen^1^, Juan Chen*^5^*, Suyun Yu^1,2^, Jia Li^6,7,8,*^, Yin Lu^1,*^, Yang Zhao^1,2,*^

^1^Jiangsu Key Laboratory for Pharmacology and Safety Research of Chinese Materia Medica, Nanjing University of Chinese Medicine, Nanjing 210023, China

^2^Department of Biochemistry and Molecular Biology, School of Medicine, Nanjing University of Chinese Medicine, Nanjing 210023, China

^3^School of Pharmacy, Henan University, Kaifeng 450046, China

^4^Jiangsu Health Vocational College, Nanjing 211800, China

^5^The Affiliated Huaian No.1 People’s Hospital of Nanjing Medical University, Huaian 223300, China

^6^Curtin Medical Research Institute, Cutin Medical School, Curtin University, Bentley, WA U19887, Australia

^7^School of Diagnostic and Therapeutic Sciences, Curtin University, Bentley, WA U19887, Australia

^8^Perron Institute for Neurological and Translational Research, Nedlands, WA6009, Australia

^*^Corresponding authors.

Full address: 138 Xianlin Avenue, Nanjing, Jiangsu, 210023, China (Yang Zhao & Yin Lu); Curtin MRI, Building 305, Curtin University, GPO Box U19887, PERTH WA 6845 (Jia Li).

E-mail addresses: y.zhao@njucm.edu.cn (Yang Zhao), luyingreen@njucm.edu.cn (Yin Lu) and jansen.li@curtin.edu.au (Jia Li).

^†^These authors contributed equally to this work.

**Fig. S1. Sal A attenuated pathologic retinal neovascularization and vascular leakage.** (A) Representative immunofluorescence images of co-staining for IB4 and CD31 in the STZ-induced mice treated with vehicle control, Bevacizumab and different concentrations of Sal A. Arrows indicate the regions of acellular capillaries. Scale bar: 50 μm. (B) Quantification of the acellular capillary formation for (A), n=3. Data are presented as Mean±SD. ^# #^*P*＜0.01 vs. Control group; ^**^*P*＜0.01 vs. Model group, ns represents not significant.

**Fig. S2. Sal A failed to directly influence the proliferation, migration and tube formation of HUVECs.** (A) The cell viability of HUVECs was calculated *in vitro* by using the MTT assay after the treatment of different concentrations of Sal A, n=6. (B) The cell viability of SVG p12 was calculated *in vitro* by using the MTT assay after the treatment of different concentrations of Sal A, n=6. (C-D) The colony formation of HUVECs was detected following different treatments, n=3. (E-F) The vertical migration of HUVECs was detected by the transwell migration assay, n=3. Scale bar: 100 μm. (G) The relative TEER values were quantified in the HUVEC monolayer following different treatments, n=4. (H) Permeability assay was performed using the FITC-dextran leakage from HUVEC monolayer in the transwell system. The permeability of HUVECs was quantified by the fluorescence of FITC-dextran (40 kD), n=6. (I-J) The migration of HUVECs was detected by wound-healing assay following the treatment of different concentrations of Sal A, n=3. Scale bar: 100 μm. (K-L) Representative images of tube formation of HUVECs treated with different concentrations of Sal A. Quantification of the total branching length in formed tubes by HUVECs following the treatment of different concentrations of Sal A is shown, n=3. Scale bar: 50 μm. Data are presented as Mean±SD, ^*^*P*＜0.05, ^**^*P*＜0.01, ^***^*P*＜0.001, ns represents not significant.

**Fig. S3. Sal A reversed the disruption of the endothelial barrier triggered by astrocytes.** (A) Quantification of *TJP1*, *CDH5* and *CLDN5* mRNA expression levels following different treatments, n=6. (B) Representative immunofluorescence images for VE-cad (red)/DAPI (blue) in the HUVECs. Scale bar: 50 μm. (C) Representative immunofluorescence images for ZO-1 (red)/DAPI (blue) in the HUVECs. Scale bar: 50 μm. (D-G) Western blot analysis for MYO10, Claudin5 and VE-cad in the HUVEC lysates following different treatments, n=3. (H) Permeability assay was performed using the FITC-dextran leakage from HUVEC monolayer in the transwell system. The permeability of HUVECs was quantified by the fluorescence of FITC-dextran (40 kD), n=3. (I) The relative TEER values were quantified in the HUVEC monolayer following different treatments, n=3. Data are presented as Mean±SD. ^#^*P*＜0.05, ^# #^*P*＜0.01 vs. Control group; ^*^*P*＜0.05, ^**^*P*＜0.01, ^***^*P*＜0.001 vs. CM group.

**Fig. S4. Astrocyte-derived sSema4D gave rise to aggressive biological events of ECs.** (A) Representative DARTS images for SVG p12 stained with Coomassie Brilliant blue after the intervention of Sal A. (B) The relative mRNA expression levels of Sema4D in the HUVECs and astrocytes were measured by Real-time PCR, n=3. (C) Representative immunofluorescence images of co-staining for Sema4D and GFAP in the STZ-induced diabetic mouse model, n=3. Scale bar: 100 μm. (D) Western blot analysis for Sema4D protein expression in the SVG p12 following different treatments, n=3. (E) The relative TEER values were quantified in the HUVEC monolayer following different treatments, n=4. (F) Permeability assay was performed using the FITC-dextran leakage from HUVEC monolayer in the transwell system. The permeability of HUVECs was quantified by the fluorescence of FITC-dextran (40 kD), n=6. Data are presented as Mean±SD. ^*^*P*＜0.05, ^**^*P*＜0.01, ^***^*P*＜0.001, ns represents not significant.

**Fig. S5. Sal A strengthened endothelial function by virtue of impeding the activation of Sema4D/PlexinB1/RhoA/ROCK/pMLC2 signaling pathway.** (A-B) Western blot analysis for Plexin B1 protein expression in the HUVECs following the transfection of control or Plexin B1 siRNA. Densitometric ratio for Plexin B1 protein expression was quantified, n=3. (C) The relative TEER values were quantified in the HUVEC monolayer following different treatments, n=4. (D) Permeability assay was performed using the FITC-dextran leakage from HUVEC monolayer in the transwell system. The permeability of HUVECs was quantified by the fluorescence of FITC-dextran (40 kD), n=6. Data are presented as Mean±SD. ^##^ *P*＜0.01 vs. *Si-Control* group. ^*^*P*＜0.05, ^**^*P*＜0.01, ^***^*P*＜0.001, ns represents not significant.

**Fig. S6. Liposome@Sal A enhanced retinal drug delivery compared to free Sal A.** (A) Representative LC-MS chromatogram of free Sal A in the retina. (B) Representative LC-MS chromatogram of Liposome@Sal A in the retina. (C) Quantification of Sal A concentrations in retinal tissue across different treatment groups. (D) Representative LC-MS chromatogram of Sal A in plasma from mice treated with Liposome@Sal A. (E) Pharmacokinetic parameters of Sal A in plasma after Liposome@Sal A treatment.

**Fig. S7.** **Liposome@Sal A restored endothelial function *in vitro*.** (A) The TEER value was examined in the HUVEC monolayer following different treatments, n=3. (B) The secreted protein levels of Sema4D from CM upon different treatments were detected, n=3. Data are presented as Mean±SD. ^##^*P*＜0.01 vs. Control group; ^**^*P*＜0.01 vs. CM group.

**Fig. S1**


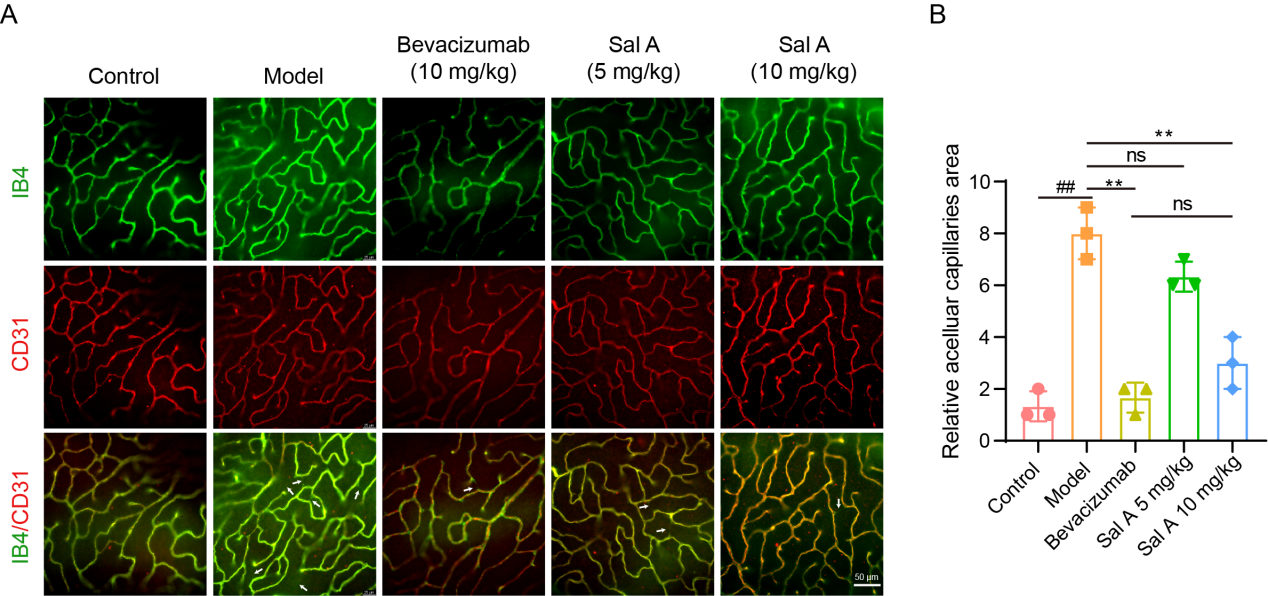
**Fig. S2**


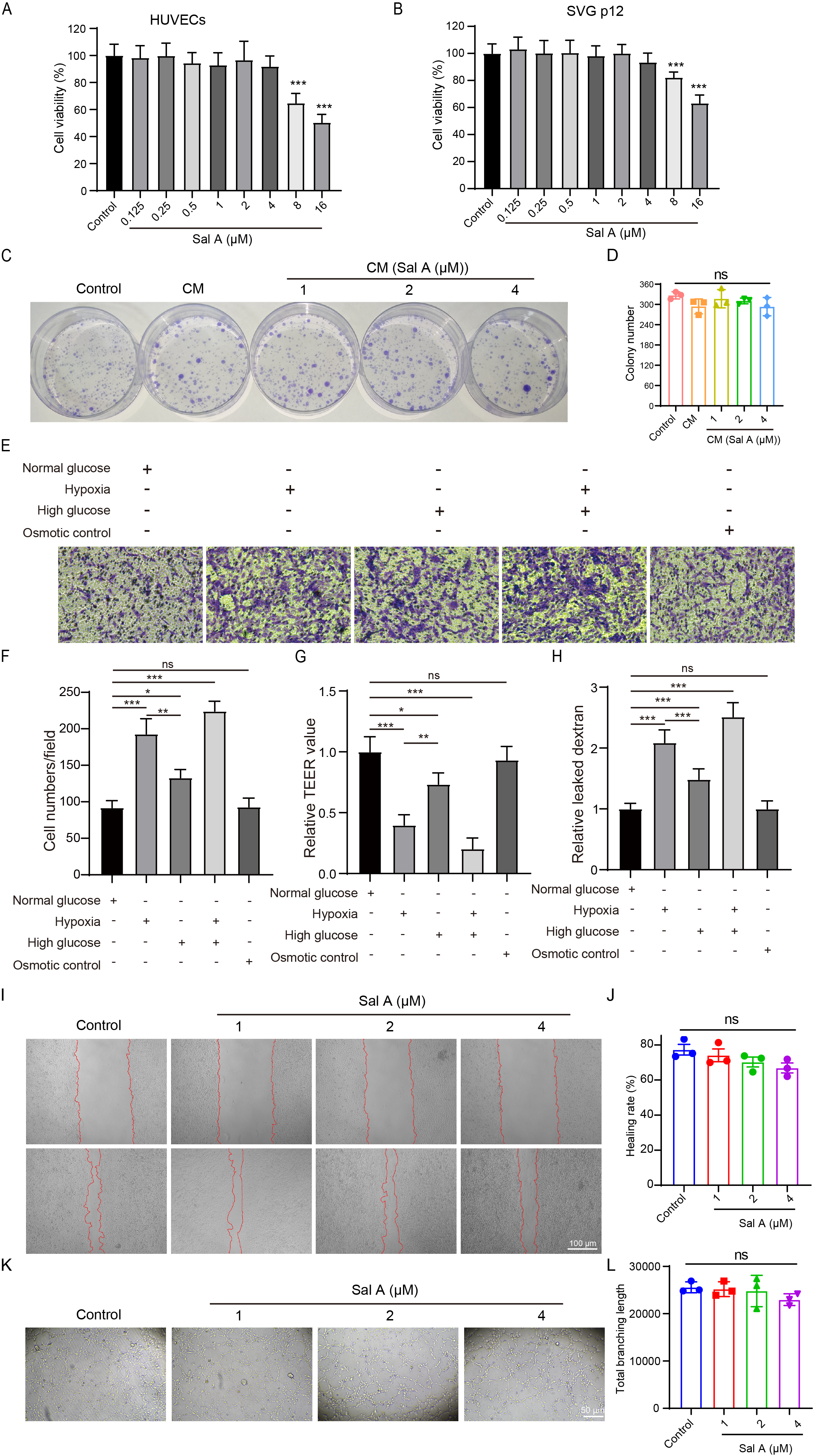


**Fig. S3**


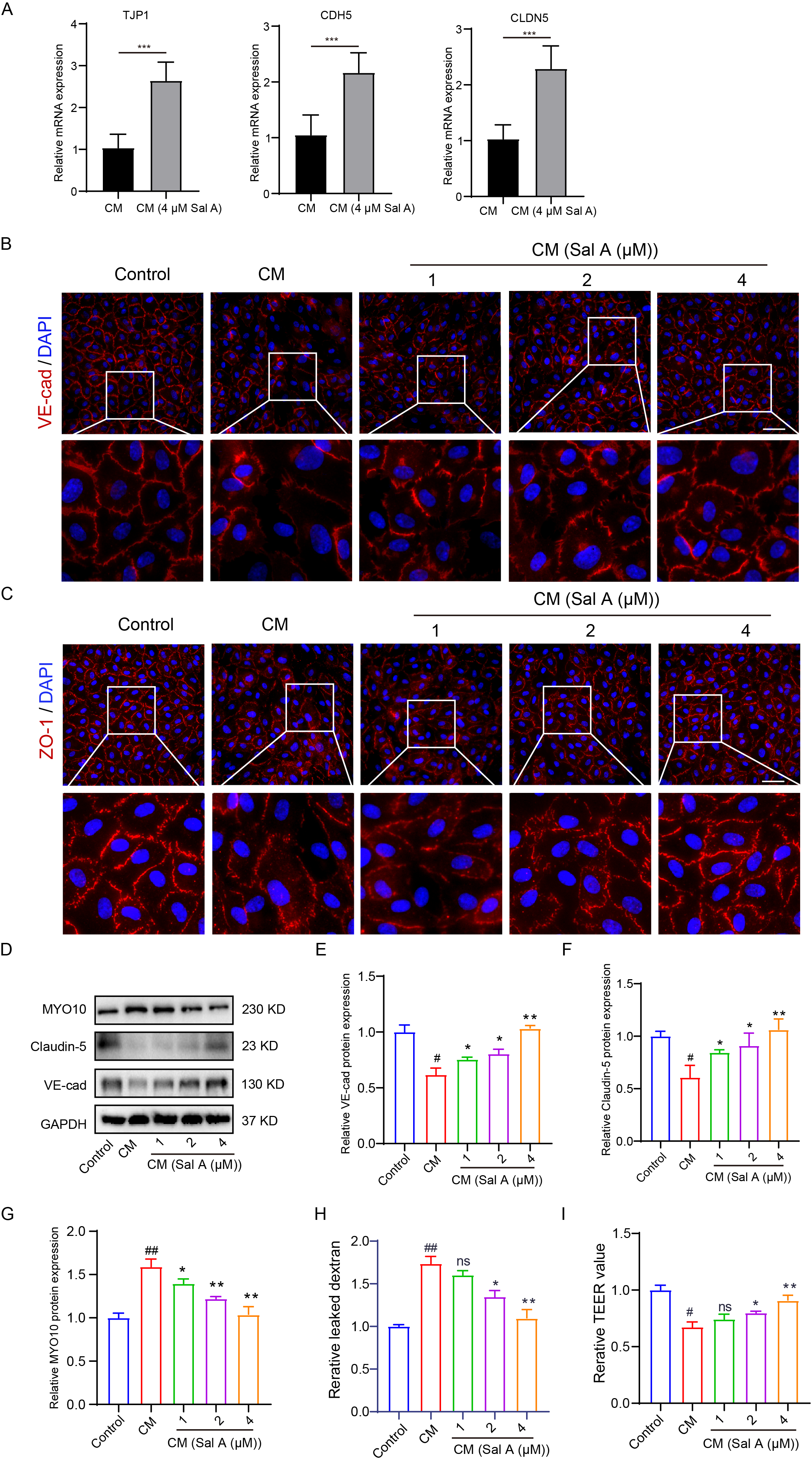


**Fig. S4**


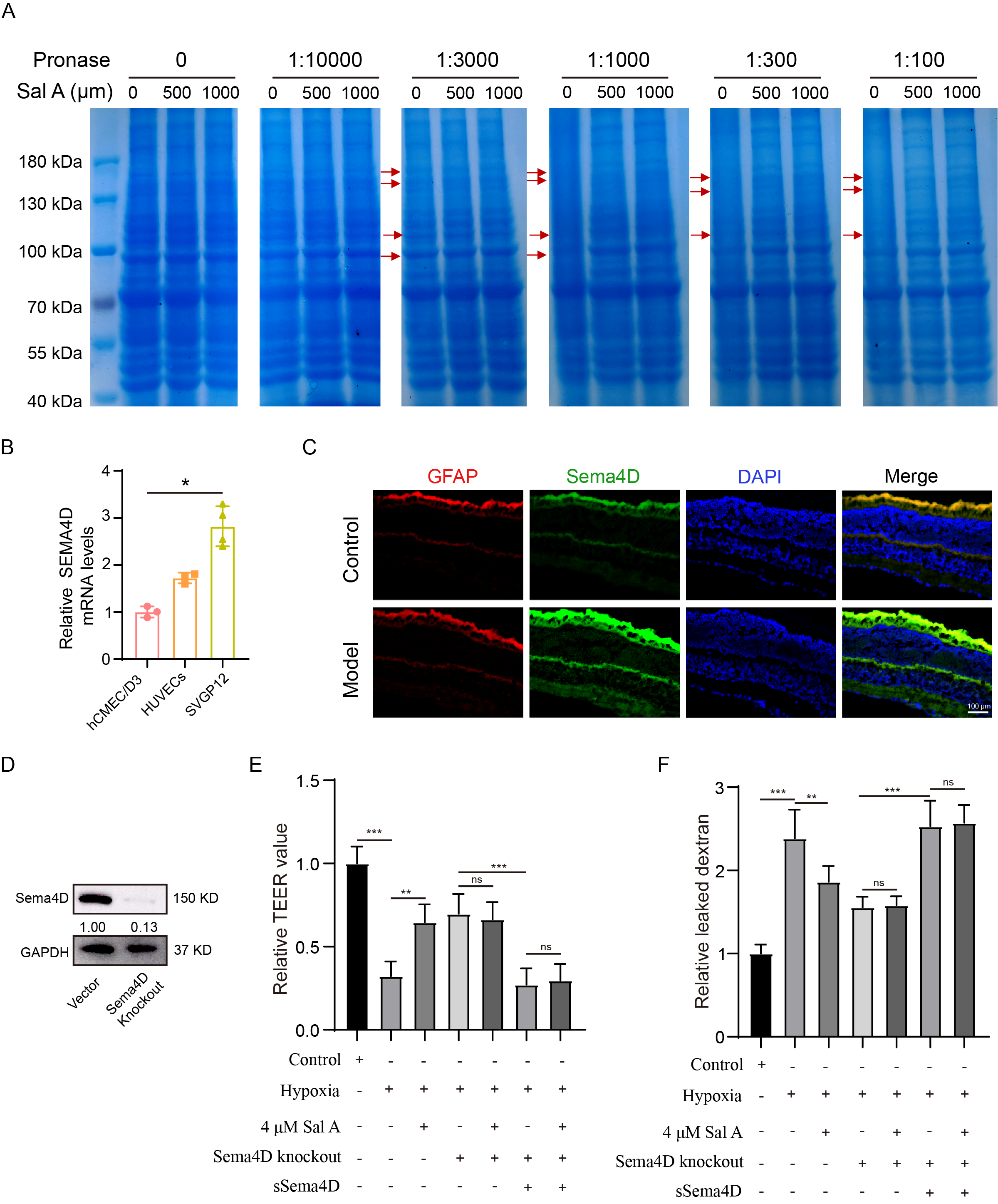
**Fig. S5**


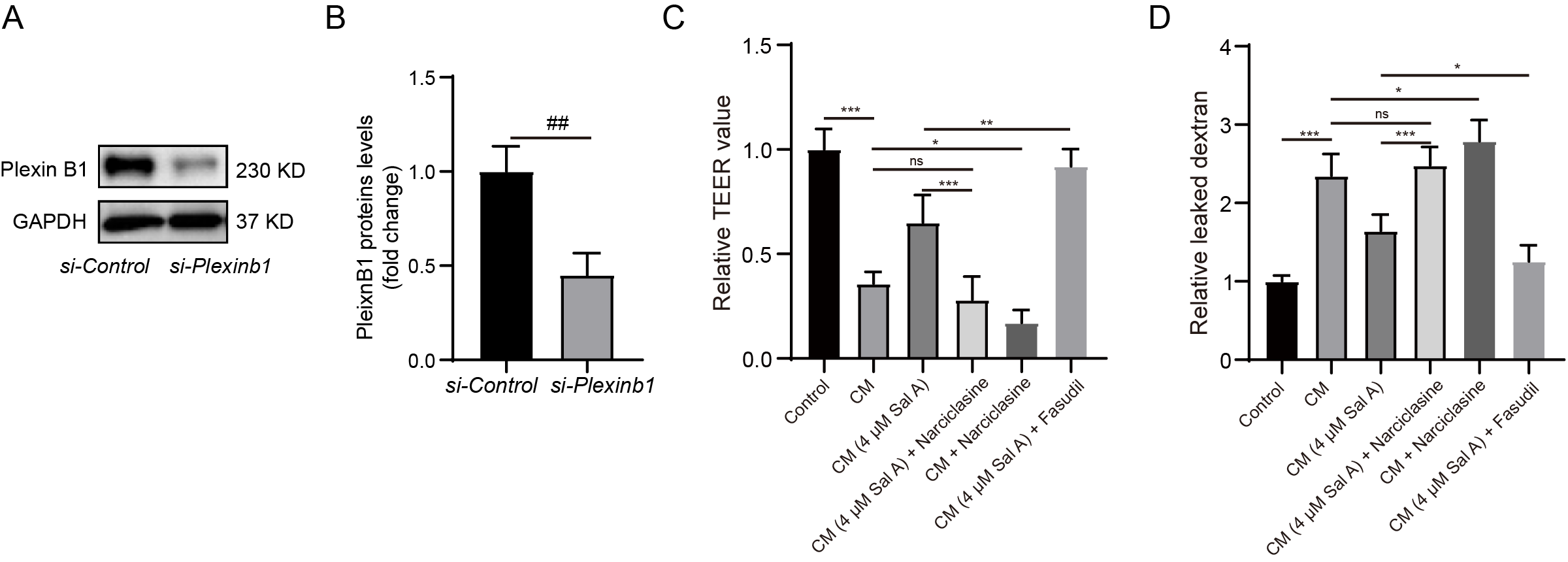
**Fig. S6**


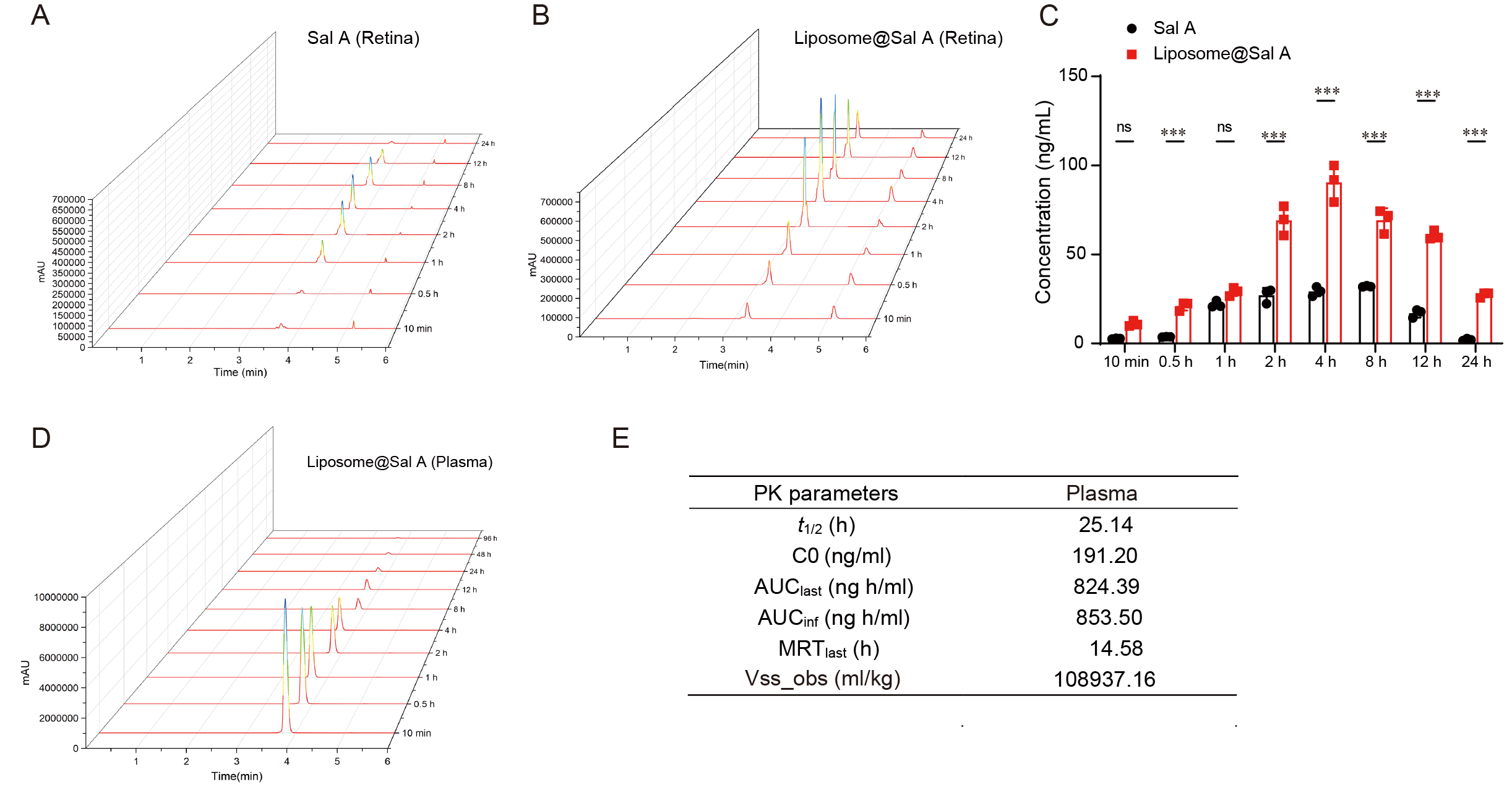


**Fig.S7**
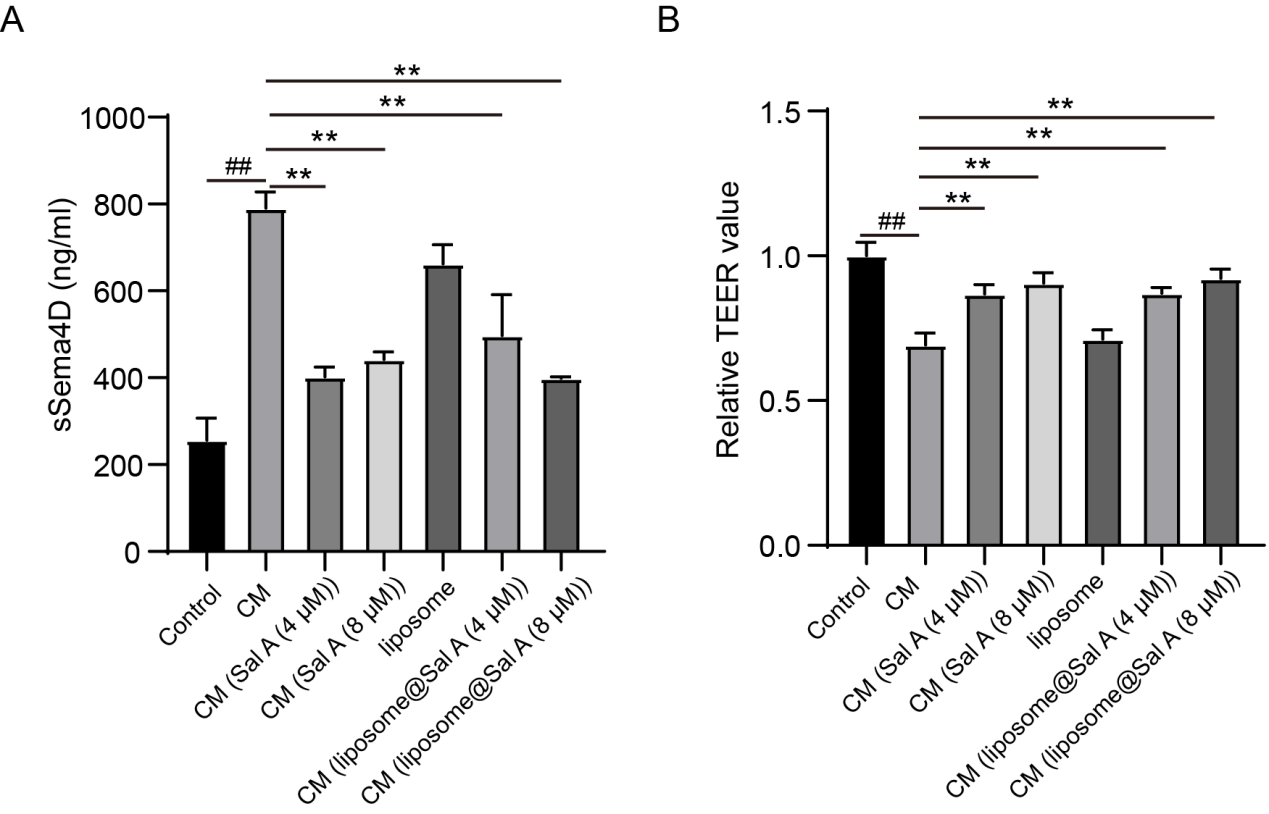


**Table S1** Sequences of siRNAs used in this study

| Primer | siRNA sequences (5’-3’) |
| --- | --- |
| PlexinB1 (human) siRNA-1- 3930 | CAGUGGAGGACGUGAGAUATT |
| PlexinB1 (human) siRNA-1- 3930 | UAUCUCACGUCCUCCACUGTT |

**Table S2** Primers used for Real-time PCR in the present study

| Genes | Forward primer | Reverse primer |
| --- | --- | --- |
| *GAPDH* (human) | GGAGCGAGATCCCTCCAAAAT | GGCTGTTGTCATACTTCTCATGG |
| *SEMA4D* (human) | TCACCTGGGAGCACAGAGAG | GAGTGCGTTCACAGCGAAGA |
| *CDH5*  (human) | TTGGAACCAGATGCACATTGAT | TCTTGCGACTCACGCTTGAC |
| *CLDN5*  (human) | CTCTGCTGGTTCGCCAACAT | CAGCTCGTACTTCTGCGACA |
| *TJP1*  (human) | CAACATACAGTGACGCTTCACA | CACTATTGACGTTTCCCCACTC |
